# Generative Artificial Intelligence in Periodontology: Application of OpenAI Models for the Classification of Periodontitis and Risk Assessment in Patients Undergoing Supportive Periodontal Care

**DOI:** 10.3390/jcm15197387

**Published:** 2026-09-23

**Authors:** Albert Camlet, Aida Kusiak, Dariusz Świetlik

**Affiliations:** 1 Department of Periodontology, Medical University of Gdansk, Skłodowskiej-Curie 3C, 80-211 Gdansk, Poland; acamlet@gumed.edu.pl (A.C.); aida.kusiak@gumed.edu.pl (A.K.); 2 Department of Biostatistics and Neural Networks, Medical University of Gdansk, Debinki 1 St., 80-211 Gdansk, Poland

**Keywords:** periodontitis classification, gingivitis, periodontal health, periodontal risk assessment, large language models, supportive periodontal care, OpenAI models, generative pre-trained transformer

## Abstract

**Background/Objectives**: Periodontitis is a chronic, inflammatory disease of teeth-supporting tissues and is highly prevalent worldwide. The current classification of periodontal diseases was introduced by the 2017 AAP/EFP World Workshop on the Classification of Periodontal and Peri-Implant Diseases and Conditions. The classification is primarily based on the staging and grading of periodontitis. Periodontal Risk Assessment (PRA), introduced by Lang and Tonetti, is an established tool for estimating the risk of periodontitis progression in patients undergoing supportive periodontal care. Large language models (LLMs) developed by OpenAI are widely investigated for potential clinical applications in medicine and dentistry. The aim of this study was to assess the agreement in PRA and stage and grade classification between clinicians and the following OpenAI models: GPT-5.2, OpenAI-o3, and OpenAI-o4-mini. **Methods**: The analysis was based on 87 medical records divided into study and control groups (57 and 30 cases, respectively). Records were reviewed for eligibility and diagnostic accuracy by two experienced clinicians. Reference diagnoses were established by consensus. A standardized prompt was used as the instruction for the OpenAI Models. The reference diagnoses were compared with the LLMs’ outputs. **Results**: LLMs differentiated periodontitis from non-periodontal cases, with sensitivity ranging from 91.2% to 94.7% and a specificity of 100%. Among correctly identified cases of periodontitis, GPT-5.2, OpenAI-o3 and OpenAI-o4-mini achieved high agreement with clinicians in stage and grade assessment (weighted κ values for stage were 0.918, 0.900, and 0.803, respectively; weighted κ values for grade were 0.857, 0.975, and 0.802, respectively), while moderate to substantial agreement was observed for PRA (weighted κ values were 0.622; 0.570; and 0.659, respectively). The analysis including all cases revealed high overall agreement between LLMs and clinicians for stage, grade and PRA, except for GPT-5.2 and OpenAI-o4-mini for grade, which presented substantial and moderate agreement, respectively. Disagreement patterns between LLMs and clinicians indicated that discrepancies occurred mostly between adjacent categories. The global comparison of three LLMs revealed statistically significant differences in agreement with clinicians’ consensus for PRA (χ^2^ = 24.01; *p* < 0.001). The paired comparison revealed statistically significant differences between OpenAI-o3 vs. OpenAI-o4-mini for staging (*p* = 0.006); GPT-5.2 vs. OpenAI-o4-mini for PRA (*p* = 0.002); and OpenAI-o3 vs. OpenAI-o4-mini for PRA (*p* < 0.001). **Conclusions**: OpenAI Models present mostly high agreement with reference diagnoses in periodontitis detection and classification and may potentially support clinical practice. However, their performance in PRA appears to be limited.

## 1. Introduction

Periodontitis is a chronic, multifactorial condition characterized by an altered host immune response induced by dysbiosis [1]. Between 1990 and 2021, the mean annual growth rate of periodontitis cases globally was 8.44%. Population growth proportionally contributes to the increase in the number of periodontitis cases. Less developed countries are more affected by periodontitis [2]. Hashim et al. emphasize the impact of periodontal disease on society, including the direct economic burden associated with disease treatment as well as the costs related to complications arising from periodontitis [3]. Periodontitis is related to other oral cavity issues, including tooth loss, pathological tooth migration, halitosis and peri-implantitis [4,5,6,7]. Moreover, the literature provides evidence of associations between periodontitis and the following systemic diseases: diabetes mellitus, cardiovascular disease, hypertension, rheumatoid arthritis and chronic kidney disease [8,9,10,11,12]. Tonetti et al. highlight the need for intensified diagnostic and therapeutic approaches in the elderly population. With the ageing of society and increased tooth survival, cases of periodontitis are expected to become more prevalent and to have a significant impact on overall health status [13].

The current definition of periodontitis was established during the 2017 World Workshop on the Classification of Periodontal and Peri-Implant Diseases and Conditions. Periodontitis is clinically diagnosed when the following criteria are met: interdental clinical attachment loss (CAL) is detected at ≥2 non-adjacent teeth or CAL ≥ 3 mm is present at the buccal or oral surface of ≥2 teeth. In such cases, probing pocket depth (PD) is >3 mm. No other local causes, including extensive restorations or vertical root fracture, should be present [14]. In addition, necrotizing periodontitis is also recognized, along with periodontitis associated with systemic diseases [15]. Necrotizing periodontitis is related to HIV infection, severe malnutrition, sleep deprivation, stress, and behavioral and environmental factors [16]. Periodontitis may manifest in association with systemic conditions such as Down syndrome, Papillon–Lefèvre syndrome, severe neutropenia, and leukocyte adhesion deficiency syndrome [17]. Current guidelines classify periodontitis using a staging and grading system. Stage defines the severity of periodontal disease and the expected treatment complexity and includes the following parameters: interdental CAL at the most affected site, radiographic bone loss (RBL), tooth loss, probing depth (PD), type of bone loss (horizontal or vertical), furcation involvement, tooth mobility and extent of disease. Grade is related to disease progression as well as expected therapeutic response and includes RBL/CAL loss in 5-year period, % RBL/age, the amount of biofilm in relation to the degree of tissue destruction, smoking and diabetes [14].

The 2017 AAP/EFP Classification poses a clinical challenge for dental professionals. Ravida et al. evaluated the agreement between 103 examiners in the classification of nine periodontal cases. The authors recorded moderate inter-examiner consistency in terms of stage, grade and extent (k values: 0.49; 0.50; and 0.51, respectively). Compared with the gold standard, the participants achieved agreement rates of 76.6%, 82%, and 84.8%, respectively [18]. In another study, authors analyzed the inter-examiner agreement among 174 participants with different levels of clinical experience. The agreement between raters was 68.7% for stage, 82.4% for grade and 75.5% for extent. The authors concluded that academic or clinical experience was insignificant in terms of the study outcomes [19]. According to Chmielewski et al., major concerns with the latest periodontitis classification include the distinction between gingivitis and stage I, as well as between stage III and stage IV. Additionally, the reliability of grade assessment could be compromised by incomplete longitudinal data. The authors suggest that training and AI-based tools may improve the efficiency of classification use in routine clinical practice [20].

A critical aspect of periodontitis management is the assessment of the patient’s overall risk of disease progression. Numerous periodontal risk tools have been proposed in the literature [21,22,23]. During supportive periodontal therapy, the risk may vary depending on periodontal parameters. A well-established tool for the systematic evaluation of progression risk is the Periodontal Risk Assessment (PRA) developed by Lang and Tonetti [24].

Large language models (LLMs) constitute a class of neural network architectures trained on large-scale datasets [25]. Most contemporary LLMs are based on the transformer architecture, which incorporates a self-attention mechanism. The concept raised by Vaswani et al. is related to assigning weights to individual elements within a sequence. As a result, the mechanism enables the modeling of complex contextual dependencies [26,27]. Consequently, these models demonstrate high effectiveness in natural language processing [28]. Furthermore, recent developments include the implementation of multimodal modules that enable the processing of images, audio and other modalities [29,30,31]. Among the most widely used large language models are those developed by OpenAI [32]. Initially, two principal development lines were distinguished: the Generative Pre-trained Transformer (GPT), a class of foundation models; and the o-series, reasoning models that employ chain-of-thought mechanisms. Following the introduction of GPT-5, progressive unification into a single model has been observed [33].

LLMs are utilized across many fields of medicine, including dermatology, pathology, neurology, pediatrics, ophthalmology, plastic surgery, and oncology [34,35,36,37,38,39,40]. LLMs have been found to be applicable in dental specialties. A recent systematic review shows that LLMs may potentially provide valuable information on oral health [41]. LLMs may also support clinical decision-making in safe antibiotic selection [42]. Researchers have demonstrated the clinical utility of LLMs in three-dimensional simulation of orthodontic treatment [43]. The multimodal capabilities of the latest models could have a significant impact on dental radiology. Liu et al. demonstrated that GPT-4o achieved high diagnostic accuracy in identifying treated teeth and dental implants. However, the model performed significantly worse in the detection of pathological lesions [44]. Another study suggests that LLMs have limited accuracy in radiographic bone height evaluation on panoramic images [45].

Considering the significant difficulties encountered by clinicians in periodontitis classification and risk evaluation, large language models may be utilized as supportive instruments in the diagnostic process. The aims of this study are the following:

- Analyze the agreement in periodontal stage and grade classification (according to 2017 AAP/EFP Classification) between OpenAI models GPT-5.2, OpenAI-o3, OpenAI-o4-mini and dental professionals.
- Analyze the agreement in Periodontal Risk Assessment between OpenAI models GPT-5.2, OpenAI-o3, and OpenAI-o4-mini and dental professionals.

## 2. Materials and Methods

### 2.1. Ethics Statement

This study was conducted in accordance with the Declaration of Helsinki and approved by the Bioethics Committee for Scientific Research of the Medical University of Gdansk on 12 December 2024 (No.: KB/517/2024). The approval was further prolonged on 15 January 2026 (No.: KB/517-9/2026). Patient consent was waived due to the retrospective nature of the study and the comprehensive anonymization of the data (no personally identifiable information available).

### 2.2. Definitions

The parameters included in the analysis are described below:

- Sex—A categorical variable representing the patient’s sex.
- Age—Patient’s age in years; a necessary parameter to assess the indirect evidence of periodontitis progression [14].
- PDmax—Maximum probing depth recorded during the examination. Probing depth is the distance between the bottom of the pocket and the free gingival margin. The parameter is measured with the standardized periodontal probe, such as PCP UNC 15 with 0.25 N force at six sites [46].
- CALmax—Maximum clinical attachment loss recorded during the examination. CAL is the distance between the bottom of the pocket and the cemento–enamel junction. The parameter is measured with the standardized periodontal probe [47].
- RBL—Radiographic bone loss at the most affected interproximal site. RBL is considered when the distance between the alveolar bone crest and the cemento–enamel junction is at least 2 mm [48].
- Tooth loss—Teeth lost due to multiple factors.
- BOP—Bleeding on probing. BOP is a direct inflammation marker [49]. Periodontal health is defined as when BOP < 10%. In the presence of 10–30% bleeding sites and PD ≤ 3 mm, localized gingivitis is diagnosed. If BOP is more than 30% and PD ≤ 3 mm, generalized gingivitis is reported [50].
- PD ≥ 5 mm—The number of residual pockets with a probing depth of ≥5 mm.
- Extent—The proportion of sites affected by periodontitis: localized (<30%) or generalized (≥30%) [51].
- Type of bone loss—Radiographic and clinical characteristics of bone loss, which may be horizontal or vertical [14].
- Furcation involvement—Bone and soft tissue loss in furcation area affecting multi-rooted teeth, measured with the standardized periodontal probe. According to the Hamp classification, class I furcation is defined in the presence of a horizontal defect <3 mm, while class II furcation is diagnosed when tissue loss exceeds 3 mm. Class III furcation is characterized when the probe passes entirely through furcation [52].
- Tooth mobility—Increased tooth mobility exceeding the physiologic range. According to the 2017 AAP/EFP periodontitis classification, tooth mobility ≥2 is distinguished as a part of secondary occlusal trauma [14].
- HbA1C—Glycated hemoglobin reflects the average glucose level in blood over approximately three months [53]. HbA1C is a vital grade modifier of the periodontitis classification [14].
- Smoking—The number of cigarettes smoked per day indicates the second grade modifier of the periodontitis classification [14].

### 2.3. Staging of Periodontitis

Parameters used to determine the stage are categorized into two principal groups: severity factors and complexity factors. The severity factors include clinical attachment loss (CAL), radiographic bone loss (RBL) and tooth loss. Both CAL and RBL are measured at interproximal surfaces. Tooth loss is considered only when related to progressive periodontitis. Therefore, cases resulting from trauma, caries complications and other factors are not included. Complexity factors include maximum probing depth, type of bone loss (horizontal or vertical), furcation involvement, ridge defects, tooth mobility and other symptoms requiring complex rehabilitation. CAL constitutes the primary criterion for staging. In the absence of reliable CAL measures, RBL is utilized in the classification. Both tooth loss and complexity factors may lead to assignment to a higher stage, despite lower CAL or RBL values. Four stages are distinguished in the classification of periodontitis. Stage I corresponds to initial periodontitis, while stage II is related to moderate periodontitis. Stage III represents periodontitis which may lead to tooth loss. Complete loss of dentition could be a complication of stage IV [14].

### 2.4. Grading of Periodontitis

The progression of periodontitis is assessed using primary criteria and grade modifiers. The primary criteria include direct evidence, which comprises RBL or CAL within the last five years. Indirect evidence is represented by the arithmetic ratio of RBL to the patient’s age. Furthermore, the proportion between the quantity of biofilm and the extent of tissue destruction is considered. Cigarette smoking and diabetes are classified as grade modifiers. The classification of periodontitis progression is divided into grade A, grade B and grade C. Grade A reflects the slow rate of progression, while grade B is assigned to moderate progression of periodontitis. Patients with a rapid rate of progression are allocated to grade C [14].

### 2.5. Periodontal Risk Assessment

Compliance during supportive periodontal care is a key factor in maintaining healthy reduced periodontium [54]. Patients with residual periodontal pockets 5 mm and above are susceptible to further progression of periodontitis. Moreover, stage IV periodontitis is related to the risk of tooth loss [55]. The Periodontal Risk Assessment is widely utilized for evaluating the risk of periodontitis progression. The tool consists of six parameters: bleeding on probing (BOP), residual periodontal pockets 5 mm and above, ratio of bone loss to the patient’s age, tooth loss, systemic and genetic factors and cigarette smoking. The risk profile is derived from the combined evaluation of all parameters and is classified into three categories: low, medium or high [24].

### 2.6. Source Data, Eligibility Criteria and Consensus Development

Textual patient charts were collected from the electronic database at the University Dental Centre. The parameters required for stage, grade and Periodontal Risk Assessment were retrieved from the charts by a trained dental professional. The data used in this study have been anonymized. Two hundred medical records, including textual patient charts, were randomly selected from the internal database. Eligibility criteria and assessment instructions were agreed upon before the analysis. The dental professional screened medical records, excluding 113 cases and retaining 87 cases. Two clinicians with 4 and 20 years of clinical experience, respectively, independently assessed the retained cases. The inter-rater agreement was calculated and the confusion matrices were constructed to characterize patterns of disagreement in periodontitis detection as well as stage, grade and PRA. Subsequently, the clinicians jointly reviewed the source records to verify the initial eligibility decisions and resolve diagnostic disagreements. The inclusion and exclusion decisions were confirmed and the diagnostic disagreements were resolved through consensus to establish the final reference standard.

Inclusion criteria for patients:

- Patients being evaluated or being treated for periodontitis at the Department of Periodontal and Oral Mucosa Diseases between January 2025 and August 2025.

Non-inclusion criteria for patients:

- Incomplete medical documentation—The absence of selected parameters used for stage, grade, periodontal risk classification or the absence of evidence that patients received treatment during supportive periodontal care.
- Patients below 18 years old.
- Patients with less than 4 teeth.
- Patients diagnosed with necrotizing periodontitis or periodontitis as the manifestation of systemic disease.
- Patients diagnosed with endo-periodontal lesions with or without root damage.
- Patients diagnosed with periodontal abscess.

Figure 1 presents the selection of patients for the study and control groups based on predefined eligibility criteria.

### 2.7. The Study Group

The study group consisted of individuals diagnosed with periodontitis who met the eligibility criteria and received non-surgical treatment. Ten individuals were classified as stage I, fourteen as stage II, twenty-seven as stage III and six as stage IV. Eight, thirty-six, and thirteen patients were classified as grades A, B, and C, respectively. Periodontal Risk Assessment (PRA) metrics were as follows: low for seven individuals, medium for twenty-six individuals and high for twenty-four individuals.

### 2.8. The Control Group

Individuals who met the eligibility criteria and exhibited either a healthy periodontium or gingivitis were included in the control group. Five patients presented with a healthy periodontium, while twenty-five patients were diagnosed with gingivitis. PRA is not applicable for the control group, as it is specific to patients with periodontal disease.

### 2.9. Analysis Conducted by OpenAI Large Language Models

The study was conducted between 20 December 2025 and 31 December 2025 with the use of GPT-5.2 (released on 11 December 2025), OpenAI-o3, and OpenAIo4-mini (both models released on 16 April 2025); OpenAI Inc., San Francisco, CA, USA. Prompts were entered using the web-based interface. Therefore, the sampling parameters, such as temperature, were neither configurable nor publicly available. Each case was analyzed only once, separately, and the conversation was deleted immediately after the response was obtained. No attachments were provided during the sessions; however, the models had access to web browsing.

The models conducted an analysis of periodontal parameters based on a standardized prompt consisting of three components. The first section provided procedural guidance for task execution, requiring the models to establish a general diagnosis, categorized as “periodontal health,” “gingivitis,” or “periodontitis”. Subsequently, in cases where periodontitis was diagnosed, the models were expected to assess the stage and the grade according to the 2017 AAP/EFP Classification [14]. Finally, the models determined the Periodontal Risk Assessment in accordance with the original guidelines developed by Lang and Tonetti [24]. The second section included periodontal parameters used by the models to establish the diagnosis. The third section listed keywords for establishing the diagnosis. Figure 2 presents the structure of the prompt, including periodontal parameters.

Figure 3 illustrates the intended classification workflow of the OpenAI models, based on the prompt content.

### 2.10. Statistical Analysis

The statistical analysis was performed using Statistica (TIBCO Software Inc., San Ramon, CA, USA; version 13.3, 2017) (https://www.tibco.com/, accessed on 20 July 2026). The comparison between the study group and the control group were performed using the Student’s *t*-test and the Mann–Whitney U for continuous variables. The Chi-square test was used for categorical variables. The sensitivity and specificity of LLMs in diagnosing periodontitis were calculated using the Clopper–Pearson method. The Clopper–Pearson analysis included data from both the study and control groups, while the agreement measures were based on the study group. Two agreement analyses were performed using the same dataset: the first was restricted to periodontitis cases correctly detected by LLMs, while the second included all cases from the study group. The agreement between LLMs and clinicians in staging, grading and PRA was assessed using exact agreement, agreement within ±1 category, Cohen’s kappa and weighted Cohen’s kappa with quadratic weights. The pre-consensus agreement between clinicians was assessed using exact agreement, Cohen’s kappa, and weighted Cohen’s kappa with quadratic weights. Confidence intervals (95% CIs) for Clopper–Pearson analysis were calculated using exact binomial methods. Confidence intervals (95% CIs) for weighted Cohen’s kappa were calculated using a nonparametric percentile bootstrap method. The overall differences among LLMs were assessed using the Friedman χ^2^ test, while pairwise comparisons were performed using the Stuart–Maxwell test with Holm correction. The significance level was set 0.05 and all estimates are presented with the corresponding 95% confidence intervals (95% CIs).

## 3. Results

### 3.1. Characteristics of the Study Group and Control Group

The study population included 57 patients aged between 18 and 86 years, while the mean age was 57.9 years (SD = 14.0). Of this population, 32 patients were women and 25 patients were men. The number of patients in the control group was 30 and the mean age was 40.1 years (SD = 11.5); 16 patients were women and 14 patients were men. Diabetes was present in 12 patients in the study group and in one patient in the control group. Four and two patients were smokers in the study group and in the control group, respectively. Statistically significant differences between groups were observed in age, PDmax, tooth loss, extent, furcation involvement, tooth mobility, and diabetes (*p* < 0.0001; *p* < 0.0001; *p* < 0.0001; *p* = 0.0017; *p* < 0.0001; *p* = 0.0046; and *p* = 0.0276, respectively). The study and control groups did not differ significantly regarding BOP, gender, smoking (*p* = 0.9929; *p* = 0.8024; and *p* = 0.9511, respectively). Certain parameters, including CALmax, RBL, PD > 5 mm and type of bone loss are solely attributable to the study group. See Table 1 and Table 2.

### 3.2. Consensus Diagnoses Established by Clinicians

Clinicians independently analyzed the cases that met the eligibility criteria. Exact agreement between clinicians in general diagnosis was observed in 78 cases. Weighted Cohen’s κ was 0.861 (95% CI: 0.781–0.941), indicating almost perfect agreement. See Table 3.

Clinician 1 and clinician 2 agreed on 53 periodontitis cases, 22 gingivitis cases and three periodontal health cases. However, disagreements between clinicians were observed in distinguishing gingivitis–periodontal health and gingivitis–periodontitis. No disagreements occurred between periodontal health and periodontitis. See Table 4.

In terms of stage assessment, clinicians achieved exact agreement in 50 cases (87.7%) and agreement within ±1 stage in all cases (100%). Cohen’s kappa was 0.821, while weighted Cohen’s kappa was 0.934 (95% CI = [0.901; 0.972]). As for grade assessment, clinicians demonstrated exact agreement in 49 cases (86.0%) and agreement within ±1 grade in 55 cases (96.5%). Cohen’s kappa was 0.760, while weighted Cohen’s kappa was 0.766 (95% CI = [0.603; 0.929]). Regarding the Periodontal Risk Assessment, clinicians showed exact agreement in 48 cases (84.2%) and agreement within ±1 grade in 56 cases (98.2%). Cohen’s kappa was 0.750, while weighted Cohen’s kappa was 0.836 (95% CI = [0.732; 0.934]). See Table 5.

Both clinicians classified six cases as stage I, 13 cases as stage II, 26 cases as stage III and five cases as stage IV. Clinician 2 reported three cases as gingivitis, while clinician 1 assessed these cases as stage I. Clinician 1 classified 1 case as gingivitis, while clinician 2 diagnosed stage I. Clinicians disagreed between stage I and stage II in one case and between stage III and stage IV in two cases. All remaining combinations had zero counts. See Table 6.

Both clinicians classified six cases as grade A, 30 cases as grade B and 13 cases as grade C. Clinician 2 reported three cases as gingivitis, while clinician 1 assessed these cases as grade A and grade B. Clinician 1 classified one case as gingivitis, while clinician 2 diagnosed grade B. Moreover, clinician 1 classified four additional cases as grade B, while clinician 2 assigned those cases to grade A and grade C. All remaining combinations had zero counts. See Table 7.

Both clinicians classified four cases as low risk, 20 cases as medium risk and 24 cases as high risk. Clinician 2 reported three cases as gingivitis, while clinician 1 assessed these cases as low risk and medium risk. Moreover, clinician 2 classified four more cases as low risk, while clinician 1 assessed these cases as gingivitis and medium risk. In another two cases, clinicians disagreed within high and medium risk categories. All remaining combinations had zero counts. See Table 8.

The study group consists of patients diagnosed with periodontitis. The control group comprised patients with gingivitis (25 cases; 83.3%) or periodontal health (five cases; 16.7%). The distribution of diagnoses varied significantly between the groups (*p* < 0.0001). Parameters stage, grade and periodontal risk are only attributable to the study group. See Table 9.

#### 3.2.1. Stage

In sum, 10 patients (17.5%) were classified to stage I periodontitis and 14 patients (24.6%) were classified to stage II periodontitis. Stage III and stage IV were assigned to 27 individuals (47.4%) and six individuals (10.5%), respectively. See Table 9.

#### 3.2.2. Grade

Clinicians classified eight patients (14.0%) to grade A, 36 patients (63.2%) to grade B and 13 patients (22.8%) to grade C. See Table 9.

#### 3.2.3. Periodontal Risk

For periodontal risk, seven patients (12.3%) were assigned to low risk and 26 patients (45.6%) were assigned to medium risk, while high risk was observed in 24 patients (42.1%). See Table 9.

### 3.3. Performance of LLMs in Periodontitis Detection

The Clopper–Pearson method was used to assess the performance of LLMs in periodontitis detection. Periodontitis was defined as a positive result, while periodontal health and gingivitis were defined as negative results. GPT-5.2 correctly identified 52 periodontitis cases and 30 non-periodontitis cases. Five cases were classified as false-negatives, while no false-positive results were noted. GPT-5.2 achieved a sensitivity of 91.2% (95% CI = [80.7%; 97.1%]). OpenAI-o3 correctly identified 54 periodontitis cases and 30 non-periodontitis cases. Three cases were classified as false-negatives, while no false-positive results were noted. OpenAI-o3 achieved a sensitivity of 94.7% (95% CI = [85.4%; 98.9%]). OpenAI-o4-mini achieved results identical to OpenAI-o3. Moreover, all three models yielded the same results in terms of specificity: 100.0% (95% CI = [88.4%; 100.0%]). See Table 10.

### 3.4. The Agreement Between LLMs and Reference Diagnoses

The agreement between OpenAI models and consensus diagnoses in periodontitis staging, grading and PRA was evaluated using exact agreement, agreement within ±1 category, Cohen’s kappa and weighted Cohen’s kappa.

#### 3.4.1. Staging

GPT-5.2 demonstrated exact agreement in 46 cases (88.5%) and agreement within ±1 stage in all cases (100%). Cohen’s kappa was 0.821, while weighted Cohen’s kappa was 0.918 (95% CI = [0.838; 0.974]), indicating almost perfect agreement. OpenAI-o3 achieved exact agreement in 47 cases (87.0%) and agreement within ±1 stage in all cases (100%). The Cohen’s kappa was 0.793, while weighted Cohen’s kappa was 0.900 (95% CI = [0.813; 0.962]). OpenAI-o4-mini yielded exact agreement in 37 cases (68.5%) and agreement within ±1 stage in 98.1% of cases. Cohen’s kappa was 0.571, while weighted Cohen’s kappa was 0.803 (95% CI = [0.680; 0.887]). See Table 11.

The overall performance of LLMs with the inclusion of all cases in the study group is presented in Table 6. GPT-5.2 demonstrated exact agreement in 46 cases (80.7%). Cohen’s kappa was 0.721, while weighted Cohen’s kappa was 0.880 (95% CI = [0.811; 0.945]); OpenAI-o3 achieved exact agreement in 47 cases (82.5%). Cohen’s kappa was 0.733, while weighted Cohen’s kappa was 0.896 (95% CI = [0.847; 0.950]); OpenAI-o4-mini yielded exact agreement in 37 cases (64.9%). Cohen’s kappa was 0.534, while weighted Cohen’s kappa was 0.810 (95% CI = [0.730; 0.884]). See Table 12.

The agreement between GPT-5.2 and clinicians was observed in five cases for stage I, 11 cases for stage II, 25 cases for stage III and five cases for stage IV. The LLM assigned five cases to gingivitis, while clinicians reported these cases in stage I and stage II. In another two cases, the disagreement was limited to stage I and stage II. GPT-5.2 assigned two cases to stage III, while clinicians diagnosed stage II and stage IV. Conversely, the LLM identified two cases as stage IV, while clinicians assessed the cases as stage III. All remaining combinations had zero counts. See Table 13.

The agreement between OpenAI-o3 and clinicians was observed in six cases for stage I, 13 cases for stage II, 26 cases for stage III and two cases for stage IV. The LLM assigned three cases to gingivitis, while clinicians reported these cases in stage I. In one case, the disagreement was limited to the stage I–stage II pair; in a second single case, the disagreement was limited to the stage II–stage III pair, and in another five cases the disagreement was limited to the stage III–stage IV pair. All remaining combinations had zero counts. See Table 14.

The agreement between OpenAI-o4-mini and clinicians was observed in six cases for stage I, 11 cases for stage II, 15 cases for stage III and five cases for stage IV. The LLM assigned two cases to gingivitis, while clinicians reported these cases in stage I. Unlike the other models, OpenAI-o4-mini classified one case as periodontal health, while clinicians classified this case as stage I. In another four cases, the disagreement was limited to the stage I–stage II pair; in one case, the disagreement was limited to the stage I–stage III pair; in a second single case, the disagreement was limited to the stage II–stage III pair; in another 11 cases, the disagreement was limited to the stage III–stage IV pair. All remaining combinations had zero counts. See Table 15.

#### 3.4.2. Grading

GPT-5.2 demonstrated exact agreement in 47 cases (90.4%) and agreement within ±1 grade in all cases (100%). Cohen’s kappa was 0.812, while weighted Cohen’s kappa was 0.857 (95% CI = [0.703; 0.963]). OpenAI-o3 achieved exact agreement in 53 cases (98.1%) and agreement within ±1 grade in all cases (100%). Cohen’s kappa was 0.966, while weighted Cohen’s kappa was 0.975 (95% CI = [0.907; 1.000]). OpenAI-o4-mini yielded exact agreement in 45 cases (83.3%) and agreement within ±1 grade in all cases (100.0%). Cohen’s kappa was 0.714, while weighted Cohen’s kappa was 0.802 (95% CI = [0.642; 0.911]). See Table 16.

GPT-5.2 demonstrated exact agreement in 47 cases (82.5%). Cohen’s kappa was 0.686, while weighted Cohen’s kappa was 0.680 (95% CI = [0.499; 0.861]). OpenAI o3 achieved exact agreement in 53 cases (93.0%). Cohen’s kappa was 0.875, while weighted Cohen’s kappa was 0.815 (95% CI = [0.640; 0.992]). OpenAI-o4-mini yielded exact agreement in 45 cases (78.9%). Cohen’s kappa was 0.649, while weighted Cohen’s kappa was 0.593 (95% CI = [0.371; 0.814]). See Table 17.

The agreement between GPT-5.2 and clinicians was observed in four cases for grade A, 31 cases for grade B and 12 cases for grade C. The LLM assigned five cases to gingivitis, while clinicians reported these cases in grade A and grade B. In another three cases, the disagreement was limited to the grade A–grade B pair; in another two cases, the disagreement was limited to the grade B–grade C pair. All remaining combinations had zero counts. See Table 18.

The agreement between OpenAI-o3 and clinicians was observed in seven cases for grade A, 33 cases for grade B and 13 cases for grade C. The LLM assigned three cases to gingivitis, while clinicians reported these cases in grade A and grade B. In one case, the disagreement was limited to the grade A–grade B pair. All remaining combinations had zero counts. See Table 19.

The agreement between OpenAI-o4-mini and clinicians was observed in eight cases for grade A, 26 cases for grade B and 11 cases for grade C. The LLM assigned two cases to gingivitis, while clinicians reported these cases in grade B. OpenAI-o4-mini classified one case as periodontal health, while clinicians diagnosed grade B. In another single case, the disagreement was limited to the grade A–grade B pair; in another eight cases the disagreement was limited to the grade B–grade C pair. All remaining combinations had zero counts. See Table 20.

#### 3.4.3. Periodontal Risk Assessment (PRA)

GPT-5.2 demonstrated exact agreement in 36 cases (69.2%), while agreement within ±1 category was 51 (98.1%). Cohen’s kappa was 0.477, while weighted Cohen’s kappa was 0.622 (95% CI = [0.402; 0.843]). OpenAI-o3 achieved exact agreement in 33 cases (61.1%), while agreement within ±1 category was 53 (98.1%). Cohen’s kappa was 0.352, while weighted Cohen’s kappa was 0.570 (95% CI = [0.385; 0.754]). OpenAI-o4-mini yielded exact agreement in 35 cases (64.8%), while agreement within ±1 category was 54 (100.0%). Cohen’s kappa was 0.430, while weighted Cohen’s kappa was 0.659 (95% CI = [0.508; 0.810]). See Table 21.

GPT-5.2 demonstrated exact agreement in 40 cases (70.2%) and agreement within ±1 grade in 56 cases (98.2%). Cohen’s kappa was 0.512, while weighted Cohen’s kappa was 0.650 (95% CI = [0.416; 0.813]). OpenAI-o3 achieved exact agreement in 35 cases (61.4%) and agreement within ±1 grade in 56 cases (98.2%). Cohen’s kappa was 0.365, while weighted Cohen’s kappa was 0.562 (95% CI = [0.358; 0.715]). OpenAI-o4-mini yielded exact agreement in 38 cases (66.7%) and agreement within ±1 grade in all cases (100.0%). Cohen’s kappa was 0.467, while weighted Cohen’s kappa was 0.680 (95% CI = [0.506; 0.809]). See Table 22.

The agreement between GPT-5.2 and clinicians was observed in six cases for low risk, 13 cases for medium risk and 21 cases for high risk. In another two cases, the disagreement was limited to the low–medium risk pair; in another one case, the disagreement was limited to the low-risk–high-risk pair; in another 14 cases, the disagreement was limited to the medium-risk–high-risk pair. All remaining combinations had zero counts. See Table 23.

The agreement between OpenAI-o3 and clinicians was observed in three cases for low risk, nine cases for medium risk and 23 cases for high risk. In another seven cases, the disagreement was limited to the low-risk–medium-risk pair; in another one case, the disagreement was limited to the low-risk–high-risk pair; in another 14 cases, the disagreement was limited to the medium-risk–high-risk pair. All remaining combinations had zero counts. See Table 24.

The agreement between OpenAI-o4-mini and clinicians was observed in six cases for low risk, 13 cases for medium risk and 19 cases for high risk. In another six cases, the disagreement was limited to the low-risk–medium-risk pair; in another 13 cases, the disagreement was limited to the medium-risk–high-risk pair. All remaining combinations had zero counts. See Table 25.

The following table, Table 21, reports the inconsistencies between periodontal diagnosis and PRA assessment. Even though in some cases, LLMs diagnosed non-periodontal categories, the PRA was assessed each time. Diagnosis–PRA inconsistencies were observed in five periodontitis cases (8.8%) for GPT-5.2 and three periodontitis cases (5.3%) for OpenAI-o3 and OpenAI-o4-mini. GPT-5.2 assigned three cases of gingivitis to medium risk and two gingivitis cases to low risk. OpenAI-o3 assigned two gingivitis cases to medium risk and 1 gingivitis case to high risk. OpenAI-o4-mini assigned two gingivitis cases to medium risk and 1 periodontal health case to low risk. See Table 26.

#### 3.4.4. Comparison Between LLMs

Global comparison between LLMs using the Friedman test (χ^2^) revealed no statistically significant differences for stage and grade. In terms of PRA, significant differences were observed (*p* < 0.001). See Table 27.

The paired comparison revealed statistically significant differences between OpenAI-o3 vs. OpenAI-o4-mini for stage (*p* = 0.006); GPT-5.2 vs. OpenAI-o4-mini for PRA (*p* = 0.002); and OpenAI-o3 vs. OpenAI-o4-mini for PRA (*p* < 0.001). See Table 28.

## 4. Discussion

Since the release of ChatGPT in 2022, the medical literature on large language models has shown a substantial increase. According to Chen et al., between January 2022 and September 2025, the increase in the number of publications follows a nearly linear trend, averaging 7.04 studies per month. The most frequently discussed topics were related to the use of LLMs in internal medicine, radiology and preventive medicine. The most used LLM was ChatGPT; however, almost 1300 studies did not provide information about the exact version of the model [56]. Dentistry is a field of science that is largely independent of medicine, and this is also reflected in the number of publications. A recent systematic review identified only 38 studies addressing the use of LLMs in dentistry [57].

Periodontology is a specialized branch of dentistry focused specifically on the diagnosis and treatment of periodontal disease. As of July 2026, we identified only four publications evaluating the use of large language models (LLMs) for the classification of periodontitis [58,59,60,61]. Eroglu et al. utilized GPT-3.5 to classify periodontitis based on 200 clinical cases. Text-only prompts were used and the outputs were compared with consensus diagnoses established by four experienced periodontists. GPT-3.5 achieved accuracy of 59.5% for staging, 50.5% for grading and 84.0% for extent. Moderate agreement was reported for staging, fair for grading and substantial for extent [58]. In another study, conducted by Ji et al., GPT-4 was used to interpret 20 clinical cases. Similarly, the authors included text-only inputs. GPT-4 responses were compared with assessments made by five non-periodontal faculty members and with the predefined reference standard. Two faculty members presented significantly better diagnostic performance than GPT-4 (*p* < 0.002); however, no significant differences were observed between the entire group of clinicians and the LLM [59]. The study by Amugo et al. demonstrated that a more recent LLM achieved better accuracy in periodontitis classification. Researchers applied GPT-5 to analyze 25 publicly available clinical cases. The results were compared with diagnoses from the source publications. Accuracy for staging was 68.0% (95% CI 48.4–82.8%), while for grading, it was 77.3% (95% CI 56.6–89.9%). Fair agreement was reported for staging and poor for grading. Despite the better overall performance of GPT-5 in periodontitis classification, the study findings should be interpreted with caution. The clinical cases used in the study were publicly available online and GPT-5 may have retrieved some of the information from the databases [60]. Among the studies discussed, Ossowska et al. compared LLM with an artificial neural network (ANN). One hundred and ten clinical cases were analyzed by GPT-4o and ANN. GPT-4o achieved accuracy of 62%, while ANN presented 85% accuracy in its responses. In terms of grade, ANN was accurate in 79% of cases, while GPT-4o was only so in 63% of cases. The differences between the two models were statistically significant (*p* < 0.0001) [61].

Several potentially confounding factors could be identified across the mentioned studies: human-prepared text-only prompts, and the absence of a control group. Across all studies, clinically relevant data was manually selected without directly incorporating periodontal charts or radiographic images. This approach could have opposing implications. A key advantage of this strategy is that directly selected parameters eliminate intermediate processing steps and allow a more direct assessment of periodontitis classification. Moreover, implementing textual cases with radiographs could complicate the determination of whether the LLM performance reflects overall diagnostic capabilities or only the image processing efficiency. Conversely, the manual preparation of case reports may introduce bias related to the individual characteristics of the person preparing the data, such as clinical experience. Another important aspect to discuss is a limitation associated with using LLMs solely for periodontitis classification without differential diagnosis. Borderline clinical scenarios could occur when a patient presents common features of periodontitis and gingivitis. For example, PD > 4 mm is one of the parameters that falls within stage I periodontitis. However, when no CAL or RBL is detected, the more appropriate diagnosis is gingivitis. PD > 4 mm does not necessarily indicate periodontium destruction and could reflect gingival swelling and pseudopocket formation [62]. To reduce the risk of bias in ambiguous clinical cases, we utilized a standardized prompt that included differential diagnosis. Three possible diagnoses were included: periodontal health, gingivitis, or periodontitis. LLMs were instructed to use the 2017 AAP/EFP Classification and Periodontal Risk Assessment only in cases of periodontitis. Additionally, unlike previous studies, we incorporated the control group consisting of patients with periodontal health or with gingivitis. Statistically significant differences between the study and control groups were observed for most parameters, supporting the clinical distinction between patients with periodontitis and patients with other diagnoses. BOP did not differ significantly between the groups (*p* = 0.9929), because high BOP values could be measured in both periodontitis and gingivitis. Smoking was also not statistically significant between the groups (*p* = 0.9511), which may be related to the small number of patients smoking cigarettes. In our study, GPT-5.2 correctly assigned 52 cases to periodontitis. Only five cases were false-negative. Both OpenAI o3 and OpenAI o4-mini classified 54 cases as periodontitis, with only three false-negative results. All three models correctly evaluated cases in the control group (30 true-negative cases), resulting in a specificity of 100% (95% CI 88.4–100.0%). Our findings indicate that OpenAI models are highly effective in distinguishing patients with periodontal conditions. Accordingly, these models could be useful for periodontitis screening in population-based studies.

Previous studies have reported staging accuracies ranging from 59.5% to 70% across different LLMs. In our study, OpenAI models achieved exact agreement of 68.5–88.5% among correctly diagnosed periodontitis cases (the overall exact agreement ranged from 64.9% to 82.5%). The lowest exact agreement for staging was observed for OpenAI-o4-mini, which is a smaller model optimized for fast and cost-efficient reasoning. However, it should be noted that agreement within ±1 stage was substantially higher and fell within a narrower range across all models from 98.1% to 100% (regarding the correctly diagnosed periodontitis cases). The disagreements between GPT-5.2 and the reference standard included the classification of stage I and stage II as gingivitis and discrepancies between stage I and stage II. The confusion matrix revealed varied misclassification patterns, particularly involving the distinction between gingivitis and early stages of periodontitis. Similarly, OpenAI-o3 and OpenAI-o4-mini classified several stage I cases as gingivitis or as gingivitis and periodontal health, respectively. However, OpenAI-o4-mini showed a significant number of discrepancies between stage III and stage IV, with a tendency to underestimate periodontitis severity. These results indicate that most classification errors occurred between adjacent categories. This may have important clinical implications such as avoidance of major misclassifications that could substantially affect treatment selection and prognostic evaluation. The weighted Cohen’s kappa for correctly diagnosed periodontitis cases demonstrated very high agreement between the models and clinicians for GPT-5.2 and OpenAI-o3 (κ = 0.918, 95% CI: 0.838–0.974; κ = 0.900, 95% CI: 0.813–0.962, respectively). OpenAI-o4-mini also showed high agreement; however, the wider confidence intervals indicated greater uncertainty (κ = 0.803, 95% CI: 0.680–0.887). The values of weighted Cohen’s kappa for the overall analysis demonstrated a comparable level of agreement between LLMs and clinicians.

In terms of grading, OpenAI models used in our study achieved high exact agreement among correctly diagnosed periodontitis cases, ranging from 83.3% to 98.1% and with 100% concordance within ±1 grade. The overall exact agreement fell within the range of 78.9–93.0%. The disagreements between LLMs and reference standard included the classification of grade A and grade B as gingivitis (particularly for GPT-5.2 and OpenAI-o3) or solely grade B as gingivitis (particularly for OpenAI-o4-mini). Disagreements between the models and clinicians generally involved adjacent categories. Interestingly, OpenAI-o4-mini presented a notable number of discrepancies between grade B and grade C. In one case, the model also classified the patient as periodontally healthy, while clinicians diagnosed grade B. Moreover, the values of weighted Cohen’s kappa for correctly diagnosed periodontitis cases indicated nearly perfect agreement between LLMs and reference diagnoses. However, the values of weighted Cohen’s kappa for the overall analysis demonstrated substantial agreement for GPT-5.2 and moderate agreement for OpenAI-o4-mini. The agreement for OpenAI-o3 was almost perfect for both analyses. For comparison, previous studies have reported LLM accuracy for grade assessment ranging from 50.5% to 77.3%. Although OpenAI-o4-mini achieved the lowest performance in stage and grade assessment among the evaluated models, the agreement with clinicians remained at least moderate. The use of a shorter and more direct prompt might potentially improve the performance of OpenAI-o4-mini, regarding the fact that the model is optimized for lower resource consumption.

The structure of the prompt significantly influences the generated responses. Prompts that are concise, precise and provide clear contextual information support the generation of more accurate responses [63]. The literature describes various prompt engineering techniques aimed at improving the efficiency of data processing, such as retrieval-augmented generation (RAG), few-shot prompting, and chain-of-thought reasoning [64]. Wang et al. classify prompts into manual and automated categories. Manual prompts are created by humans and are highly precise but also time-consuming. In contrast, automated prompts are generated by specialized algorithms and are applied in more complex processes [65]. In clinical applications of LLMs, manual prompting plays a more important role than automated prompting. Zero-shot prompting is a technique that refers to providing the instructions without any specific examples. One-shot and few-shot techniques involve prompts that contain at least one example illustrating the given task. Prompts that incorporate examples lead to more reliable responses [66]. Consistent with the literature, we utilized a zero-shot prompting strategy, which allows verification of whether the LLM could perform a task without in-context learning.

Analysis of LLM performance in Periodontal Risk Assessment revealed a partial reversal of the previously observed trend. OpenAI-o4-mini achieved the highest concordance with reference diagnoses (κ = 0.680, 95% CI: 0.506–0.809; κ = 0.659, 95% CI: 0.508–0.810, regarding the overall agreement) and all the answers were accurate within ±1 category. The overall agreement for all models ranged from 61.4% to 70.2%, whereas concordance within ±1 category was substantially higher, ranging from 98.2% to 100%. The exact agreement among correctly detected periodontitis cases ranged from 61.1% to 69.2%. PRA classification comprises only three categories; therefore, the high agreement within adjacent categories may not accurately reflect the clinical implications. In this context, the exact agreement, which was substantially lower, is more representative for LLMs’ performance. The analysis of discrepancies between LLMs and the clinicians revealed a strong tendency to assign medium-risk cases to higher category. However, no low-risk cases were classified as high-risk by the models. Among the remaining clinical cases, GPT-5.2 presented four disagreements with clinicians involving adjacent risk categories, and one involving non-adjacent risk categories. OpenAI-o3 presented seven cases of disagreement involving adjacent risk categories and one case involving non-adjacent risk categories. OpenAI-o4-mini presented disagreements exclusively within the same risk categories. We observed internal inconsistencies in the responses of LLMs regarding the periodontal risk, which could be partially related to the methodology of this study. The inconsistencies between general diagnosis and risk assessment in LLMs’ responses involved the low-risk–gingivitis pair and the medium-risk–gingivitis pair for GPT-5.2; medium-risk–gingivitis pair and high-risk–gingivitis pair for OpenAI-o3; and the medium-risk–gingivitis pair and low-risk–periodontal health pair for OpenAI-o4-mini. PRA is a clinical tool intended for use in patients with signs of periodontitis, such as the presence of residual pockets >5 mm and alveolar bone loss. These parameters are not applicable to patients diagnosed with gingivitis or periodontal health. Despite assigning such diagnoses, the models simultaneously performed PRA, which depicts a lack of coherence. This error was observed in the general-purpose model and those using chain-of-thought reasoning, indicating that it may arise from the prompt structure or a shared underlying mechanism. A discrepancy in even one adjacent category may have substantial implications for treatment recommendations, including the management of modifiable risk factors and the frequency of follow-up appointments. Therefore, the exact agreement and weighted Cohen’s kappa are more relevant for evaluating the potential clinical utility of LLMs. PRA not only estimates the risk of further periodontitis progression but also supports clinical decision-making by identifying the need for intervention regarding oral hygiene, smoking and glycemic control. Accordingly, PRA serves as an indicator of periodontal therapy needs over time, as well as of changes in health-related behaviors. Nevertheless, the literature highlights new potential areas that may be relevant to periodontology. Butera et al. conducted a systematic literature review evaluating the use of probiotics as an adjunct to non-surgical periodontal therapy. The authors concluded that there is limited evidence supporting improvements in periodontal parameters over a 3-month period. However, the results were based on heterogeneous, short-term studies [67]. Scribante highlighted the development of minimally invasive periodontal therapies targeting the oral microbiota, including ozone therapy and the use of probiotics, paraprobiotics and postbiotics. The aim of these approaches is to shift the microbial balance from pathogenic toward commensal bacteria and reduce inflammation [68].

The global comparison performed with the Friedman test showed the statistical differences between LLMs in Periodontal Risk Assessment (*p* < 0.001). Moreover, we used the Stuart–Maxwell test with Holm correction to compare LLM pairs. Regarding PRA, OpenAI-o3 significantly more frequently assigned higher risk categories than GPT-5.2 and OpenAI-o4-mini (*p* = 0.002; *p* < 0.001, respectively). In terms of grade, OpenAI-o4-mini significantly more frequently assigned stage III and less frequently assigned stage IV, relative to OpenAI-o3.

Our findings should be also interpreted in the context of inter-rater agreement between human examiners, as methodological differences between studies may substantially affect the generalizability of the results. In our study, the two experienced clinicians’ independent assessments were analyzed before consensus was reached. In terms of general diagnosis, clinicians agreed upon 78 cases out of 87. The disagreement in the five cases was related to periodontal health and gingivitis. The main difference between those diagnoses is BOP parameter, which may be sometimes misclassified. Clinicians reported different diagnoses in four more cases including periodontitis and gingivitis. No disagreements involved the periodontal health–periodontitis pair. Despite the minor differences, the agreement in periodontitis detection was almost perfect. With respect to staging, disagreements between clinicians occurred between adjacent categories: gingivitis and stage I, and between stage III and stage IV. No discrepancies were observed between early stages and advanced stages of periodontitis. Regarding the grade and PRA, isolated disagreements were observed, including non-adjacent categories. Inter-clinician agreement was almost perfect for staging and PRA, with a narrow confidence interval (κ = 0.934, 95%CI: 0.901; 0.72; κ = 0.766, 95%CI: 0.603–0.929, respectively), while the agreement for grading was substantial with wider confidence intervals (κ = 0.836, 95%CI: 0.732–0.934). Oh et al. evaluated agreement in periodontitis classification between individuals with and without a periodontal background. Participants evaluated clinical cases based on photographic and radiographic images, as well as periodontal charts. The data were not specifically curated for the study and therefore more closely reflected routine clinical practice. Fair agreement was observed between the two groups. However, among the group of individuals with periodontal experience, the level of agreement was significantly higher (*p* = 0.05) [69]. In another study, authors compared the agreement between dental students, periodontology students and orthodontic students. Participants established diagnoses based on unprocessed clinical data, including diagnostic images and periodontal charts. The agreement between groups was fair, while periodontal students presented the highest within-group agreement (κ = 0.24 vs. κ = 0.55, respectively) [70].

The prompt used in our study had a structured, three-part design. The first section provided instructions for classifying the patients into one of three possible diagnoses: periodontal health, gingivitis, or periodontitis. The subsequent instruction was conditional, specifying that the staging and grading classification should apply only to patients with periodontitis. In addition, the model was instructed to determine PRA, which by default concerned individuals with periodontitis. The complexity and interdependence of these instructions may substantially influence the model’s effectiveness. The second section presented the parameters required to fulfill the instructions from the first section. The parameters were listed in separate rows and the units were specified in parentheses. The third section presented predefined keywords for describing stage, grade and periodontal risk. By using a limited and well-defined nomenclature, we reduced the likelihood of generating invalid outputs. In summary, the advantages of this prompt include a clear division into sections, a precisely defined objective with reference to medical guidelines, a standardized response nomenclature and the complete set of information required to generate the response. However, no examples were included in the instructions section (zero-shot prompting). Delivering samples that illustrate the topic has been reported to enhance the performance of LLMs [71,72].

Another important aspect in interpreting the results is the multifactorial structure of the 2017 AAP/EFP Classification. When assessing the stage, both the severity and complexity parameters are considered. The presence of a single parameter indicating a higher stage is sufficient to upshift the diagnosis. A similar rule applies to the grade, where parameters are divided into primary criteria and grade modifiers. The primary criteria are further divided into direct evidence and indirect evidence. LLMs tend to underperform in compositional reasoning tasks that require solving multi-step problems [73,74,75].

The literature includes numerous publications addressing staging, grading and PRA, such as those regarding official guidelines, reviews and research articles. It remains unclear which reference datasets were utilized by the models to accomplish the task. The prompt used in our study specified which classifications should be used; however, no articles were directly provided. This approach may be described as instructional prompting. Nevertheless, more effective prompting strategies are described in the literature. Retrieval augmented generation (RAG) is a strategy in which relevant contextual data are retrieved and incorporated into the prompt, enabling the model to generate more precise responses [76].

Other factors that may reduce the accuracy of the responses are different reference ranges for the PRA and the grading system. This applies to smoking status, diabetes and the percentage of bone loss relative to age. For instance, within the PRA, the %bone loss/age ratio is categorized as ≤0.5, 0.5–1.0, and >1.0 for low, moderate, and high risk, respectively. However, the grading system defines the corresponding thresholds as <0.25, 0.25–1.0, and >1.0, respectively. LLMs tend to exhibit higher error rates with an increasing number of options. This phenomenon is known as boundary ambiguity [77].

The increasing popularity of LLMs is associated with significant ethical and environmental concerns. A significant risk for sensitive data processed by LLMs is the potential for its reuse during the generation of outputs [78]. All data entered into LLMs should be anonymized and must not contain any information that could lead to the identification of a person. Large language models are trained on immense data volumes, which results in substantial energy and water consumption [79].

Considering the availability of established tools for assessing periodontitis progression requiring fewer resources, such as the PRA (perio-tools.com/pra), the replacement of these methods with LLMs is not justified. Nevertheless, LLMs represent a promising tool for the classification of periodontitis. For less experienced general practitioners, those tools may provide an opportunity to obtain accurate diagnoses. Conversely, the uncritical reliance on LLMs in routine clinical practice may impair clinician’s ability to make independent decisions and reduce motivation in continuing education.

We have identified important limitations of our study:

1. Small sample size—The analysis included periodontal parameters obtained from 57 cases in the study group and 30 cases in the control group. Importantly, the diagnosis of periodontitis relies on the assessment of both stage and grade. Consequently, certain stage and grade combinations may occur less frequently than others, for example, stage 4, grade A periodontitis. The sample used in our publication is representative of the distribution of periodontitis among patients presenting to our clinic. However, the issue of the limited number of individual combinations remains unresolved.
2. Lack of external validation—External validation is commonly used to assess the generalizability of LLMs in performing specific tasks [80,81,82]. Our research strategy was to assess the agreement between OpenAI models and a reference standard established by two experts. However, external validation, including processing an independent dataset, was not utilized.
3. The use of general-purpose LLMs only—GPT-5.2, OpenAI-o3 and OpenAI-o4-mini are general-purpose models that are applicable to non-specific tasks including text, audio and image modification. Given their ability to process open-source data, such as scientific papers, guidelines and clinical trials, these models may be applicable in clinical practice. Additionally, the literature includes examples of domain-specific LLMs that were fine-tuned using biomedical data [83,84,85]. A comparison of general-purpose and domain-specific models may enable researchers to assess whether fine-tuning improves the accuracy of periodontitis classification.
4. The use of a single prompting strategy—The prompt used in our study was based on a zero-shot strategy. A comparison of different prompting techniques against the reference standard would provide additional information.
5. Manual data preparation—The models evaluated data retrieved from the database that was prepared in a standardized manner by the trained dental practitioner. The models did not directly analyze radiographic images or parameters derived from periodontal charts, which is an important limitation when assessing the applicability of LLMs in routine clinical practice.
6. The absence of borderline cases—The characteristics of the study and control group presented typical cases of patients. Our study does not permit an assessment of the reliability of the models in ambiguous clinical scenarios.
7. Single analysis of each case—The prompting strategy involved a single analysis of each case. Therefore, it was not possible to assess response variability as a function of the time intervals between analyses or the number of repeated evaluations.

## 5. Conclusions

1. Large language models demonstrated high performance in distinguishing periodontal cases from non-periodontal cases. The findings suggest that LLMs may be effective in screening and epidemiological research. However, we evaluated a relatively small dataset in our study. Further studies involving larger and externally validated populations are needed to confirm the generalizability of the findings. In the analysis restricted to cases correctly detected as periodontitis, large language models achieved very high agreement with clinicians’ consensus in stage and grade assessment. Moreover, the agreement within adjacent categories was nearly 100%. The overall agreement between LLMs and clinicians remained almost perfect regarding staging; however, GPT-5.2 and OpenAI-o4-mini presented substantial and moderate agreement with clinicians in terms of grading, respectively. LLMs performance may be comparable to expert consensus diagnoses in non-ambiguous cases. However, in routine clinical practice, LLMs’ agreement with clinicians may differ.
2. In the context of PRA, LLMs demonstrated moderate to substantial agreement with clinicians, although the agreement within one category was nearly 100% (among the cases correctly detected as periodontitis and in the overall study population). However, PRA includes only three categories; therefore, the results should be interpreted cautiously. The clinical utility of LLMs for periodontitis progression appears to be limited.
3. Most disagreements between LLMs and clinicians involved adjacent categories, with a minor proportion of discrepancies exceeding one category.
4. The overall comparison of three LLMs revealed no statistically significant differences in agreement with clinicians’ consensus for staging and grading, while a significant difference was observed for PRA (*p* < 0.001). The paired comparison showed statistically significant differences for OpenAI-o3 vs. OpenAI-o4-mini in stage assessment (*p* = 0.006) and for OpenAI-o3 vs. GPT-5.2 (*p* = 0.002) and vs. OpenAI-o4-mini (*p* < 0.001), both in PRA.

We used general-purpose LLMs to evaluate standardized clinical cases curated by a trained dental professional in terms of staging, grading and PRA. The models demonstrated a high level of agreement with the reference standard established by consensus between two experts. However, these findings are methodologically limited by the study design, including the use of a relatively small, single-center, retrospective sample. Moreover, the stage and grade were assessed only in cases which were diagnosed as periodontitis. Therefore, our results should be interpreted as proof-of-concept evidence rather than validation of clinical utility. Further investigation of the clinical potential of LLMs in periodontology should involve multicenter studies across heterogenous populations, supported by externally validated datasets. Further research is needed to evaluate the applicability of LLMs in the diagnosis of periodontitis. However, it should be noted that new models are constantly being released and replacing older versions. This has a significant impact on the ability to compare models and form conclusions. There is a need for systematic reviews and meta-analyses to understand the development of LLMs in periodontology and other fields of medicine.

## Figures and Tables

**Figure 1:**
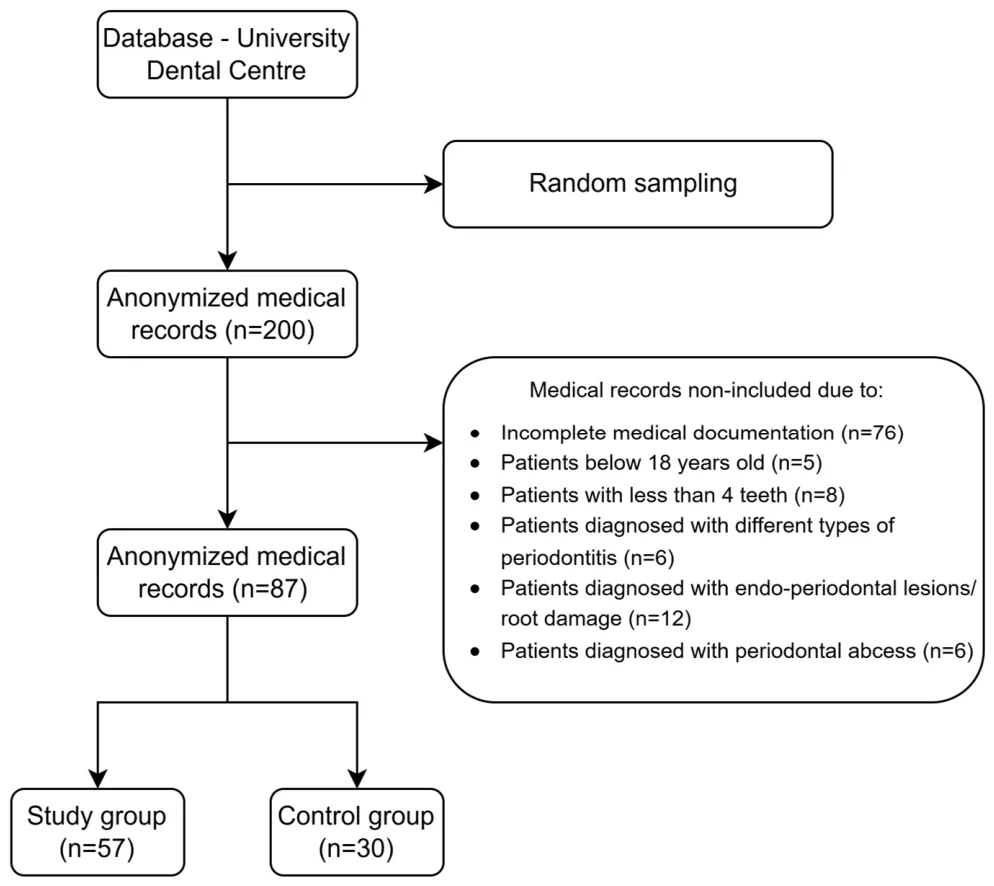
Flow chart illustrating the selection of patients, regarding the inclusion and non-inclusion criteria.

**Figure 2:**
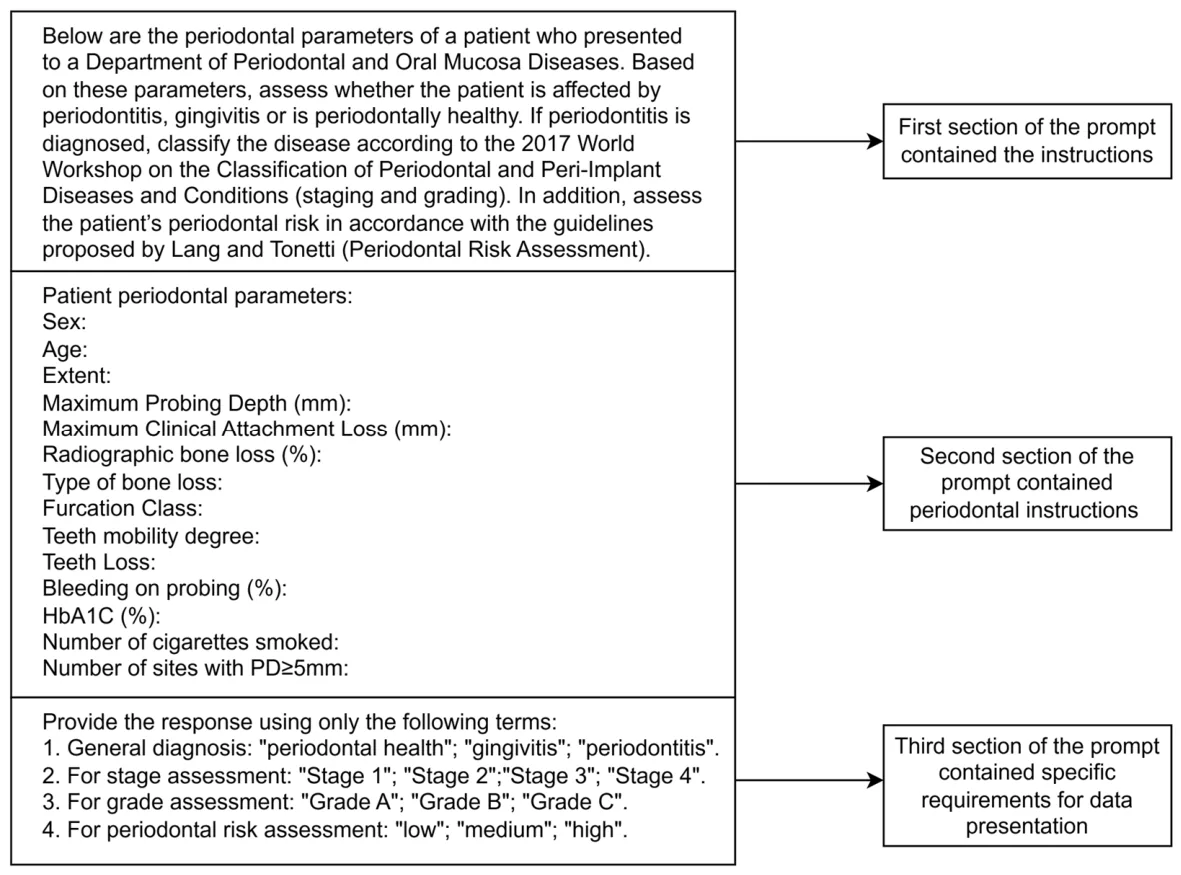
Representative prompt structured into three main sections.

**Figure 3:**
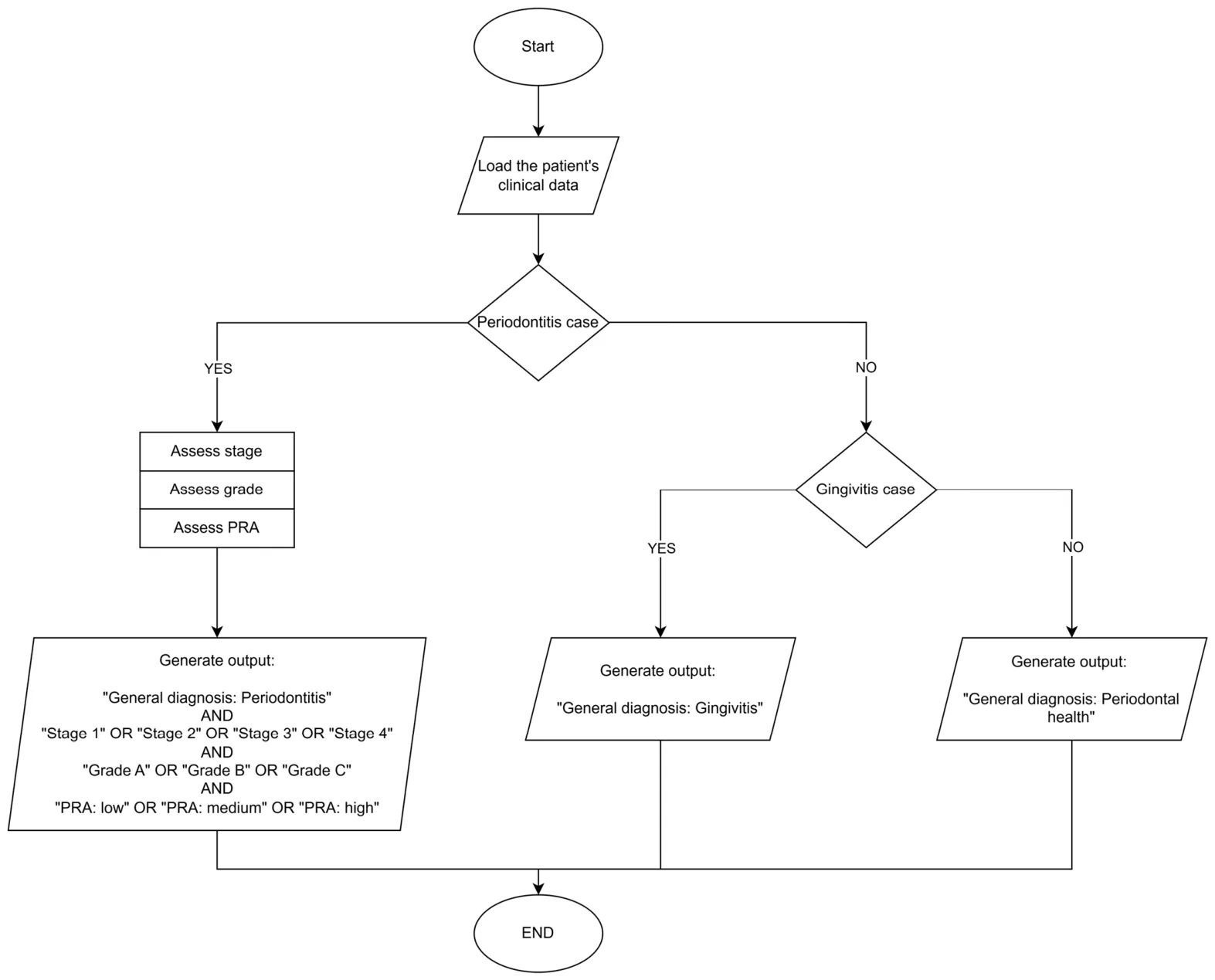
Schematic representation of the expected OpenAI models workflow based on the provided prompt.

**Table 1:** Characteristics of the study and control groups.

	Study Group (*n* = 57)	Control Group (*n* = 30)	*p*-Value
**Age (years)**			<0.0001 ^1^
Mean (SD)	57.9 (14.0)	40.1 (11.5)	
Range	18.0–86.0	22.0–61.0	
Median (IQR)	58.0 (20.0)	38.5 (20.0)	
95% CI	[54.2; 61.6]	[35.8; 44.4]	
**PDmax (mm)**			<0.0001 ^2^
Mean (SD)	5,6 (2.1)	2.9 (0.8)	
Range	2.0–12.0	2.0–4.0	
Median (IQR)	5.0 (2.0)	3.0 (2.0)	
95% CI	[5.1; 6.2]	[2.6; 3.2]	
**CALmax (mm)**		--	--
Mean (SD)	4.8 (2.9)		
Range	1.0–15.0		
Median (IQR)	4.0 (5.0)		
95% CI	[4.0; 5.5]		
**RBL (%)**		--	--
Mean (SD)	30.4 (14.7)		
Range	10.0–72.0		
Median (IQR)	31.0 (23.0)		
95% CI	[26.5; 34.3]		
**Tooth loss (*n*)**			<0.0001 ^2^
Mean (SD)	5.0 (4.7)	1.3 (1.6)	
Range	0.0–22.0	0.0–6.0	
Median (IQR)	5.0 (6.0)	1.0 (2.0)	
95% CI	[3.8; 6.3]	[0.7; 1.9]	
**BOP (%)**			0.9929 ^2^
Mean (SD)	41.3 (28.4)	41.5 (30.3)	
Range	0.0–100.0	0.0–100.0	
Median (IQR)	33.0 (40.0)	38.0 (53.0)	
95% CI	[33.8; 48.9]	[30.2; 52.8]	
**PD ≥ 5 mm (*n*)**		--	--
Mean (SD)	6.6 (7.1)		
Range	0.0–28.0		
Median (IQR)	5.0 (10.0)		
95% CI	[4.7; 8.4]		

SD—standard deviation; IQR—interquartile range; CI—confidence interval; PDmax—maximum pocket depth; CALmax—maximum clinical attachment loss; RBL—radiographic bone loss; BOP—bleeding on probing; ^1^ t-Student; ^2^ Mann–Whitney U.

**Table 2:** Characteristics of the study and control groups (continued).

	Study Group (*n* = 57)	Control Group (*n* = 30)	*p*-Value
**Sex (*n*)**			0.8024 ^1^
Female	32 (56.1%)	16 (53.3%)	
Male	25 (43.9%)	14 (46.7%)	
**Extent (*n*)**			0.0017 ^1^
Generalized	49 (86.0%)	19 (63.3%)	
Localized	8 (14.0%)	5 (16.7%)	
None	0 (0.0%)	6 (20.0%)	
**Type of bone loss (*n*)**		--	--
Horizontal	52 (91.2%)		
Mixed	5 (8.8%)		
None	0 (0.0%)		
**Furcation involvement (*n*)**			<0.0001 ^1^
	23 (41.8%)	0 (0.0%)	
**Tooth mobility (*n*)**			0.0046 ^1^
	13 (22.8%)	0 (0.0%)	
**Diabetes (*n*)**			0.0276 ^1^
	12 (21.1%)	1 (3.3%)	
**Smoking (*n*)**			0.9511 ^1^
	4 (7.0%)	2 (6.7%)	

^1^ Chi-square.

**Table 3:** Pre-consensus agreement between clinicians for periodontitis detection.

Parameter	Number of Cases	Exact Agreement	Cohen’s κ	Weighted Cohen’s κ (95% CI)
General diagnosis	87	78 (89.7%)	0.794	0.861 (0.781–0.941)

CI—confidence interval.

**Table 4:** Confusion matrix for pre-consensus periodontitis detection (clinician 1 vs. clinician 2).

Clinician 1	Clinician 2		
	Periodontal Health	Gingivitis	Periodontitis
**Periodontal health**	3	2	0
**Gingivitis**	3	22	1
**Periodontitis**	0	3	53

**Table 5:** Pre-consensus agreement between clinicians for stage, grade and periodontal risk classification.

Parameter	Number of Cases	Exact Agreement, *n* (%)	Agreement within ±1 Category (%), *n*	Cohen’s κ	Weighted Cohen’s κ (95% CI)
Stage	57	50 (87.7%)	57 (100%)	0.821	0.934 (0.901–0.972)
Grade	57	49 (86.0%)	55 (96.5%)	0.760	0.766 (0.603–0.929)
Periodontal Risk Assessment	57	48 (84.2%)	56 (98.2%)	0.750	0.836 (0.732–0.934)

CI—confidence interval.

**Table 6:** Confusion matrix for pre-consensus stage classification (clinician 1 vs. clinician 2).

Clinician 1	Clinician 2					
	Periodontal Health	Gingivitis	Stage 1	Stage 2	Stage 3	Stage 4
**Periodontal health**	0	0	0	0	0	0
**Gingivitis**	0	0	1	0	0	0
**Stage 1**	0	3	6	0	0	0
**Stage 2**	0	0	1	13	0	0
**Stage 3**	0	0	0	0	26	2
**Stage 4**	0	0	0	0	0	5

**Table 7:** Confusion matrix for pre-consensus grade classification (clinician 1 vs. clinician 2).

Clinician 1	Clinician 2				
	Periodontal Health	Gingivitis	Grade A	Grade B	Grade C
**Periodontal health**	0	0	0	0	0
**Gingivitis**	0	0	0	1	0
**Grade A**	0	2	6	0	0
**Grade B**	0	1	2	30	2
**Grade C**	0	0	0	0	13

**Table 8:** Confusion matrix for pre-consensus Periodontal Risk Assessment (clinician 1 vs. clinician 2).

Clinician 1	Clinician 2				
	Periodontal Health	Gingivitis	Low	Medium	High
**Periodontal health**	0	0	0	0	0
**Gingivitis**	0	0	1	0	0
**Low**	0	2	4	0	0
**Medium**	0	1	3	20	2
**High**	0	0	0	0	24

**Table 9:** Distribution of diagnoses in the study and control groups.

	Study Group (*n* = 57)	Control Group (*n* = 30)	*p*-Value
**Periodontitis (*n*)**			<0.0001
Periodontal health	0 (0.0%)	5 (16.7%)	
Gingivitis	0 (0.0%)	25 (83.3%)	
Periodontitis	57 (100.0%)	0 (0.0%)	
**Stage (*n*)**		--	--
1	10 (17.5%)		
2	14 (24.6%)		
3	27 (47.4%)		
4	6 (10.5%)		
**Grade (*n*)**		--	--
A	8 (14.0%)		
B	36 (63.2%)		
C	13 (22.8%)		
**Periodontal risk (*n*)**		--	--
Low	7 (12.3%)		
Medium	26 (45.6%)		
High	24 (42.1%)		

**Table 10:** Performance of LLMs in periodontitis detection.

Model	TP	FN	TN	FP	Sensitivity % (95% CI)	Specificity % (95% CI)
GPT-5.2	52	5	30	0	91.2% (80.7–97.1)	100.0% (88.4–100.0)
OpenAI-o3	54	3	30	0	94.7% (85.4–98.9)	100.0% (88.4–100.0)
OpenAI-o4-mini	54	3	30	0	94.7% (85.4–98.9)	100.0% (88.4–100.0)

TP—true-positive; FN—false-negative; TN—true-negative; FP—false-positive; CI—confidence interval.

**Table 11:** Agreement between LLMs and clinicians for stage assessment among correctly detected periodontitis cases.

Model	Number of Cases	Exact Agreement, *n* (%)	Agreement within ±1 Category (%), *n*	Cohen’s κ	Weighted Cohen’s κ (95% CI)
GPT-5.2	52	46 (88.5%)	52 (100.0%)	0.821	0.918 (0.838–0.974)
OpenAI-o3	54	47 (87.0%)	54 (100.0%)	0.793	0.900 (0.813–0.962)
OpenAI-o4-mini	54	37 (68.5%)	53 (98.1%)	0.571	0.803 (0.680–0.887)

CI—confidence interval.

**Table 12:** Overall agreement between LLMs and clinicians for stage assessment.

Model	Number of Cases	Exact Agreement, *n* (%)	Cohen’s κ	Weighted Cohen’s κ (95% CI)
GPT-5.2	57	46 (80.7%)	0.721	0.880 (0.811–0.945)
OpenAI-o3	57	47 (82.5%)	0.733	0.896 (0.847–0.950)
OpenAI-o4-mini	57	37 (64.9%)	0.534	0.810 (0.730–0.884)

CI—confidence interval.

**Table 13:** Confusion matrix for stage assessment (GTP-5.2 vs. clinicians).

Clinicians	GPT-5.2					
	Periodontal Health	Gingivitis	Stage I	Stage II	Stage III	Stage IV
**Stage I**	0	4	5	1	0	0
**Stage II**	0	1	1	11	1	0
**Stage III**	0	0	0	0	25	2
**Stage IV**	0	0	0	0	1	5

**Table 14:** Confusion matrix for stage assessment (OpenAI-o3 vs. clinicians).

Clinicians	OpenAI-o3					
	Periodontal Health	Gingivitis	Stage I	Stage II	Stage III	Stage IV
**Stage I**	0	3	6	1	0	0
**Stage II**	0	0	0	13	1	0
**Stage III**	0	0	0	0	26	1
**Stage IV**	0	0	0	0	4	2

**Table 15:** Confusion matrix for stage assessment (GPT-o4-mini vs. clinicians).

Clinicians	GPT-o4-Mini					
	Periodontal Health	Gingivitis	Stage I	Stage II	Stage III	Stage IV
**Stage I**	1	2	6	1	0	0
**Stage II**	0	0	3	11	0	0
**Stage III**	0	0	1	1	15	10
**Stage IV**	0	0	0	0	1	5

**Table 16:** Agreement between LLMs and clinicians for grade assessment among correctly detected periodontitis cases.

Model	Number of Cases	Exact Agreement, *n* (%)	Agreement within ±1 Category (%), *n*	Cohen’s κ	Weighted Cohen’s κ (95% CI)
GPT-5.2	52	47 (90.4%)	52 (100.0%)	0.812	0.857 (0.703–0.963)
OpenAI-o3	54	53 (98.1%)	54 (100.0%)	0.966	0.975 (0.907–1.000)
OpenAI-o4-mini	54	45 (83.3%)	54 (100.0%)	0.714	0.802 (0.642–0.911)

CI—confidence interval.

**Table 17:** Overall agreement between LLMs and clinicians for grade assessment.

Model	Number of Cases	Exact Agreement, *n* (%)	Cohen’s κ (95% CI)	Weighted Cohen’s κ (95% CI)
GPT-5.2	57	47 (82.5%)	0.686	0.680 (0.499–0.861)
OpenAI-o3	57	53 (93.0%)	0.875	0.815 (0.640–0.992)
OpenAI-o4-mini	57	45 (78.9%)	0.649	0.593 (0.371–0.814)

CI—confidence interval.

**Table 18:** Confusion matrix for grade assessment (GPT-5.2 vs. clinicians).

Clinicians	GPT-5.2				
	Periodontal Health	Gingivitis	Grade A	Grade B	Grade C
**Grade A**	0	2	4	2	0
**Grade B**	0	3	1	31	1
**Grade C**	0	0	0	1	12

**Table 19:** Confusion matrix for grade assessment (OpenAI-o3 vs. clinicians).

Clinicians	OpenAI-o3				
	Periodontal Health	Gingivitis	Grade A	Grade B	Grade C
**Grade A**	0	1	7	0	0
**Grade B**	0	2	1	33	0
**Grade C**	0	0	0	0	13

**Table 20:** Confusion matrix for grade assessment (OpenAI-o4-mini vs. clinicians).

Clinicians	OpenAI-o4-Mini				
	Periodontal Health	Gingivitis	Grade A	Grade B	Grade C
**Grade A**	0	0	8	0	0
**Grade B**	1	2	1	26	6
**Grade C**	0	0	0	2	11

**Table 21:** Agreement between LLMs and clinicians for PRA among correctly detected periodontitis cases.

Model	Number of Cases	Exact Agreement, *n* (%)	Agreement within ±1 Category (%), *n*	Cohen’s κ	Weighted Cohen’s κ (95% CI)
GPT-5.2	52	36 (69.2%)	51 (98.1%)	0.477	0.622 (0.402–0.843)
OpenAI-o3	54	33 (61.1%)	53 (98.1%)	0.352	0.570 (0.385–0.754)
OpenAI-o4-mini	54	35 (64.8%)	54 (100.0%)	0.430	0.659 (0.508–0.810)

CI—confidence interval.

**Table 22:** Overall agreement between LLMs and clinicians for PRA.

Model	Number of Cases	Exact Agreement, *n* (%)	Agreement within ±1 Category (%), *n*	Cohen’s κ	Weighted Cohen’s κ (95% CI)
GPT-5.2	57	40 (70.2%)	56 (98.2%)	0.512	0.650 (95% CI: 0.416–0.813)
OpenAI-o3	57	35 (61.4%)	56 (98.2%)	0.365	0.562 (95% CI: 0.358–0.715)
OpenAI-o4-mini	57	38 (66.7%)	57 (100.0%)	0.467	0.680 (95% CI: 0.506–0.809)

CI—confidence interval.

**Table 23:** Confusion matrix for PRA (GPT-5.2 vs. clinicians).

Clinicians	GPT-5.2		
	Low	Medium	High
**Low**	6	1	0
**Medium**	1	13	12
**High**	1	2	21

**Table 24:** Confusion matrix for PRA (OpenAI-o3 vs. clinicians).

Clinicians	OpenAI-o3		
	Low	Medium	High
**Low**	3	4	0
**Medium**	3	9	14
**High**	1	0	23

**Table 25:** Confusion matrix for PRA (OpenAI-o4-mini vs. clinicians).

Clinicians	OpenAI-o4-Mini		
	Low	Medium	High
**Low**	6	1	0
**Medium**	5	13	8
**High**	0	5	19

**Table 26:** Frequency of general diagnosis–PRA inconsistencies in LLMs’ responses.

Model	Periodontal Diagnosis	Low Risk (*n*)	Medium Risk (*n*)	High Risk (*n*)
GPT-5.2	Gingivitis	2	3	0
OpenAI-o3	Gingivitis	0	2	1
OpenAI-4-mini	Gingivitis	0	2	0
OpenAI-o4-mini	Periodontal health	1	0	0

**Table 27:** Global comparison of stage, grade and PRA between LLMs.

Parameter	Friedman χ^2^	*p*-Value
Stage	3.36	0.186
Grade	1.50	0.472
PRA	24.01	<0.001

PRA—Periodontal Risk Assessment.

**Table 28:** Paired comparison of stage, grade and PRA between LLMs.

Model Comparison	Stuart–Maxwell Test with Holm Correction, *p*-Value		
	Stage	Grade	PRA
GPT-5.2 vs. OpenAI-o3	>0.05	>0.05	0.002
GPT-5.2 vs. OpenAI-o4-mini	>0.05	>0.05	>0.05
OpenAI-o3 vs. OpenAI-o4-mini	0.006	>0.05	<0.001

## Data Availability

Representative examples supporting the study procedures are provided in the manuscript. Due to ethical considerations, further data are available upon reasonable request.

## Abbreviations

The following abbreviations are used in this manuscript:

LLMs	Large language models
AAP	American Academy of Periodontology
EFP	European Federation of Periodontology
GPT	Generative pre-trained transformer
PD	Probing depth
PDmax	Maximum probing depth
CAL	Clinical attachment loss
CALmax	Maximum clinical attachment loss
RBL	Radiographic bone loss
BOP	Bleeding on probing
PRA	Periodontal Risk Assessment
HbA1C	Glycosylated hemoglobin A1C
RAG	Retrieval-augmented generation
IRB	Institutional review board
ANN	Artificial neural network
SD	Standard deviation
IQR	Interquartile range
CI	Confidence interval
TP	True-positive
FN	False-negative
TN	True-negative
FP	False-positive

## References

[1] Mehrnia N., Van Dyke T.E. (2026). Microbial Dysbiosis and Immune Dysregulation in Periodontitis and Peri-Implantitis. Front. Cell. Infect. Microbiol..

[2] Fu H., Li X., Zhang R., Zhu J., Wang X. (2025). Global Burden of Periodontal Diseases among the Working-Age Population from 1990–2021: Results from the Global Burden of Disease Study 2021. BMC Public Health.

[3] Hashim N.T., Babiker R., Padmanabhan V., Ahmed A.T., Chaitanya N.C.S.K., Mohammed R., Priya S.P., Ahmed A., El Bahra S., Islam M.S. (2025). The Global Burden of Periodontal Disease: A Narrative Review on Unveiling Socioeconomic and Health Challenges. Int. J. Environ. Res. Public Health.

[4] Ravidà A., Qazi M., Troiano G., Saleh M.H.A., Greenwell H., Kornman K., Wang H. (2020). Using Periodontal Staging and Grading System as a Prognostic Factor for Future Tooth Loss: A Long-term Retrospective Study. J. Periodontol..

[5] Zasčiurinskienė E., Rastokaitė L., Lindsten R., Basevičienė N., Šidlauskas A. (2023). Malocclusions, Pathologic Tooth Migration, and the Need for Orthodontic Treatment in Subjects with Stage III–IV Periodontitis. A Cross-Sectional Study. Eur. J. Orthod..

[6] Silva M.F., Cademartori M.G., Leite F.R.M., López R., Demarco F.F., Nascimento G.G. (2017). Is Periodontitis Associated with Halitosis? A Systematic Review and Meta-regression Analysis. J. Clin. Periodontol..

[7] Serroni M., Borgnakke W.S., Romano L., Balice G., Paolantonio M., Saleh M.H.A., Ravidà A. (2024). History of Periodontitis as a Risk Factor for Implant Failure and Incidence of Peri-implantitis: A Systematic Review, Meta-analysis, and Trial Sequential Analysis of Prospective Cohort Studies. Clin. Implant Dent. Rel. Res..

[8] Stöhr J., Barbaresko J., Neuenschwander M., Schlesinger S. (2021). Bidirectional Association between Periodontal Disease and Diabetes Mellitus: A Systematic Review and Meta-Analysis of Cohort Studies. Sci. Rep..

[9] Leng Y., Hu Q., Ling Q., Yao X., Liu M., Chen J., Yan Z., Dai Q. (2023). Periodontal Disease Is Associated with the Risk of Cardiovascular Disease Independent of Sex: A Meta-Analysis. Front. Cardiovasc. Med..

[10] Muñoz Aguilera E., Suvan J., Buti J., Czesnikiewicz-Guzik M., Barbosa Ribeiro A., Orlandi M., Guzik T.J., Hingorani A.D., Nart J., D’Aiuto F. (2020). Periodontitis Is Associated with Hypertension: A Systematic Review and Meta-Analysis. Cardiovasc. Res..

[11] Fuggle N.R., Smith T.O., Kaul A., Sofat N. (2016). Hand to Mouth: A Systematic Review and Meta-Analysis of the Association between Rheumatoid Arthritis and Periodontitis. Front. Immunol..

[12] Chapple I.L.C., Hirschfeld J., Cockwell P., Dietrich T., Sharma P. (2025). Interplay between Periodontitis and Chronic Kidney Disease. Nat. Rev. Nephrol..

[13] Tonetti M.S., Bottenberg P., Conrads G., Eickholz P., Heasman P., Huysmans M., López R., Madianos P., Müller F., Needleman I. (2017). Dental Caries and Periodontal Diseases in the Ageing Population: Call to Action to Protect and Enhance Oral Health and Well-Being as an Essential Component of Healthy Ageing–Consensus Report of Group 4 of the Joint EFP / ORCA Workshop on the Boundaries Between Caries and Periodontal Diseases. J. Clin. Periodontol..

[14] Papapanou P.N., Sanz M., Buduneli N., Dietrich T., Feres M., Fine D.H., Flemmig T.F., Garcia R., Giannobile W.V., Graziani F. (2018). Periodontitis: Consensus Report of Workgroup 2 of the 2017 World Workshop on the Classification of Periodontal and Peri-Implant Diseases and Conditions. J. Periodontol..

[15] Tonetti M.S., Greenwell H., Kornman K.S. (2018). Staging and Grading of Periodontitis: Framework and Proposal of a New Classification and Case Definition. J. Periodontol..

[16] Herrera D., Retamal-Valdes B., Alonso B., Feres M. (2018). Acute Periodontal Lesions (Periodontal Abscesses and Necrotizing Periodontal Diseases) and Endo-periodontal Lesions. J. Clin. Periodontol..

[17] Jepsen S., Caton J.G., Albandar J.M., Bissada N.F., Bouchard P., Cortellini P., Demirel K., De Sanctis M., Ercoli C., Fan J. (2018). Periodontal Manifestations of Systemic Diseases and Developmental and Acquired Conditions: Consensus Report of Workgroup 3 of the 2017 World Workshop on the Classification of Periodontal and Peri-Implant Diseases and Conditions. J. Periodontol..

[18] Ravidà A., Travan S., Saleh M.H.A., Greenwell H., Papapanou P.N., Sanz M., Tonetti M., Wang H., Kornman K. (2021). Agreement among International Periodontal Experts Using the 2017 World Workshop Classification of Periodontitis. J. Periodontol..

[19] Abrahamian L., Pascual-LaRocca A., Barallat L., Valles C., Herrera D., Sanz M., Nart J., Figuero E. (2022). Intra- and Inter-examiner Reliability in Classifying Periodontitis According to the 2018 Classification of Periodontal Diseases. J. Clin. Periodontol..

[20] Chmielewski M., Pilloni A., Cuozzo A., D’Albis G., D’Elia G., Papi P., Marini L. (2025). The 2018 Classification of Periodontitis: Challenges from Clinical Perspective. Dent. J..

[21] Page R.C., Krall E.A., Martin J., Mancl L., Garcia R.I. (2002). Validity and Accuracy of a Risk Calculator in Predicting Periodontal Disease. J. Am. Dent. Assoc..

[22] Leininger M., Tenenbaum H., Davideau J. (2010). Modified Periodontal Risk Assessment Score: Long-term Predictive Value of Treatment Outcomes. A Retrospective Study. J. Clin. Periodontol..

[23] Teich S.T. (2013). Risk Assessment-Based Individualized Treatment (RABIT): A Comprehensive Approach to Dental Patient Recall. J. Dent. Educ..

[24] Lang N.P., Tonetti M.S. (2003). Periodontal Risk Assessment (PRA) for Patients in Supportive Periodontal Therapy (SPT). Oral Health Prev. Dent..

[25] Vrdoljak J., Boban Z., Vilović M., Kumrić M., Božić J. (2025). A Review of Large Language Models in Medical Education, Clinical Decision Support, and Healthcare Administration. Healthcare.

[26] Vaswani A., Shazeer N., Parmar N., Uszkoreit J., Jones L., Gomez A.N., Kaiser L., Polosukhin I. (2023). Attention Is All You Need. arXiv.

[27] Belhaouari S.B., Kraidia I. (2025). Efficient Self-Attention with Smart Pruning for Sustainable Large Language Models. Sci. Rep..

[28] Ren M. (2024). Advancements and Applications of Large Language Models in Natural Language Processing: A Comprehensive Review. Appl. Comput. Eng..

[29] Huang D., Yan C., Li Q., Peng X. (2024). From Large Language Models to Large Multimodal Models: A Literature Review. Appl. Sci..

[30] AlSaad R., Abd-alrazaq A., Boughorbel S., Ahmed A., Renault M.-A., Damseh R., Sheikh J. (2024). Multimodal Large Language Models in Health Care: Applications, Challenges, and Future Outlook. J. Med. Internet Res..

[31] Meskó B. (2023). The Impact of Multimodal Large Language Models on Health Care’s Future. J. Med. Internet Res..

[32] Shah N.H., Entwistle D., Pfeffer M.A. (2023). Creation and Adoption of Large Language Models in Medicine. JAMA.

[33] Kumar K., Vasimalla Y., Kumar S. (2025). Fundamentals of Generative AI. Generative AI for Photonic Sensing.

[34] Goktas P., Grzybowski A. (2024). Assessing the Impact of ChatGPT in Dermatology: A Comprehensive Rapid Review. J. Clin. Med..

[35] Bahadir C.D., Akar G.B., Sabuncu M.R. (2025). LLM-Generated Rewrite and Context Modulation for Enhanced Vision Language Models in Digital Pathology. Proceedings of the 2025 IEEE/CVF Winter Conference on Applications of Computer Vision (WACV).

[36] Moura L., Jones D.T., Sheikh I.S., Murphy S., Kalfin M., Kummer B.R., Weathers A.L., Grinspan Z.M., Silsbee H.M., Jones L.K. (2024). Implications of Large Language Models for Quality and Efficiency of Neurologic Care: Emerging Issues in Neurology. Neurology.

[37] Huang T., Tse G., Pageler N.M., Bannett Y. (2026). Large Language Models Using Clinical Text in Pediatrics: A Scoping Review. JAMA Netw. Open.

[38] Kwon M., Jang K.J., Baek S.J., Han Y.S., Choi H., Lee I., Kim J.H. (2025). Ophtimus-V2-Tx: A Compact Domain-Specific LLM for Ophthalmic Diagnosis and Treatment Planning. Sci. Rep..

[39] Mohapatra D.P., Thiruvoth F.M., Tripathy S., Rajan S., Vathulya M., Lakshmi P., Singh V.K., Haq A.U. (2023). Leveraging Large Language Models (LLM) for the Plastic Surgery Resident Training: Do They Have a Role?. Indian J. Plast. Surg..

[40] Carl N., Schramm F., Haggenmüller S., Kather J.N., Hetz M.J., Wies C., Michel M.S., Wessels F., Brinker T.J. (2024). Large Language Model Use in Clinical Oncology. npj Precis. Onc..

[41] Zhang R., Pan Y., Liu Y., Deng Y., Pow E.H.N. (2025). Leveraging Large Language Models for Patient Instructions in Dentistry—A Systematic Review and Meta-analysis. J. Prosthodont..

[42] Han Z., Ge J., Li C. (2025). Knowledge-Guided Large Language Model for Automatic Pediatric Dental Record Understanding and Safe Antibiotic Recommendation. arXiv.

[43] Wang Z., Hong J., Zhu Z. (2024). Deep Tooth LLM: Neural Trajectory Optimization for Tooth Alignment. Int. J. Multidiscip. Res. Growth Eval..

[44] Liu Z., Ai Q.Y.H., Yeung A.W.K., Tanaka R., Nalley A., Hung K.F. (2025). Performance of a Vision-Language Model in Detecting Common Dental Conditions on Panoramic Radiographs Using Different Tooth Numbering Systems. Diagnostics.

[45] Camlet A., Kusiak A., Ossowska A., Świetlik D. (2025). Advances in Periodontal Diagnostics: Application of MultiModal Language Models in Visual Interpretation of Panoramic Radiographs. Diagnostics.

[46] Heitz-Mayfield L.J.A. (2024). Conventional Diagnostic Criteria for Periodontal Diseases (Plaque-induced Gingivitis and Periodontitis). Periodontology 2000.

[47] Chuai Y., Dai B., Liu X., Hu M., Wang Y., Zhang H. (2023). Association of Vitamin K, Fibre Intake and Progression of Periodontal Attachment Loss in American Adults. BMC Oral Health.

[48] Helmi M.F., Huang H., Goodson J.M., Hasturk H., Tavares M., Natto Z.S. (2019). Prevalence of Periodontitis and Alveolar Bone Loss in a Patient Population at Harvard School of Dental Medicine. BMC Oral Health.

[49] Newbrun E. (1996). Indices to Measure Gingival Bleeding. J. Periodontol..

[50] Chapple I.L.C., Mealey B.L., Van Dyke T.E., Bartold P.M., Dommisch H., Eickholz P., Geisinger M.L., Genco R.J., Glogauer M., Goldstein M. (2018). Periodontal Health and Gingival Diseases and Conditions on an Intact and a Reduced Periodontium: Consensus Report of Workgroup 1 of the 2017 World Workshop on the Classification of Periodontal and Peri-Implant Diseases and Conditions. J. Periodontol..

[51] Holde G.E., Bunæs D.F., Jönsson B. (2025). Comparison Between the 2017 EFP / AAP and CDC / AAP Case Definitions on Periodontal Status and Their Association With Risk Factors in an Adult Norwegian Population—Insights From the Tromsø Study: Tromsø7. Int. J. Dent. Hyg..

[52] Pilloni A., Rojas M.A. (2018). Furcation Involvement Classification: A Comprehensive Review and a New System Proposal. Dent. J..

[53] Sherwani S.I., Khan H.A., Ekhzaimy A., Masood A., Sakharkar M.K. (2016). Significance of HbA1c Test in Diagnosis and Prognosis of Diabetic Patients. Biomark. Insights.

[54] Echeverría J.J., Echeverría A., Caffesse R.G. (2019). Adherence to Supportive Periodontal Treatment. Periodontology 2000.

[55] Siow D.S.F., Goh E.X.J., Ong M.M.A., Preshaw P.M. (2023). Risk Factors for Tooth Loss and Progression of Periodontitis in Patients Undergoing Periodontal Maintenance Therapy. J. Clin. Periodontol..

[56] Chen S.F., Alyakin A., Seas A., Yang E., Choi J.J., Lee J.V., Chen A.L., Warman P.I., Bitolas R.T., Steele R.J. (2026). LLM-Assisted Systematic Review of Large Language Models in Clinical Medicine. Nat. Med..

[57] Kaplan T.T., Akyol G.E. (2026). The Role of Large Language Models in Dental Diagnosis, Decision-Making, and Communication: A Systematic Review. Jpn. Dent. Sci. Rev..

[58] Tastan Eroglu Z., Babayigit O., Ozkan Sen D., Ucan Yarkac F. (2024). Performance of ChatGPT in Classifying Periodontitis According to the 2018 Classification of Periodontal Diseases. Clin. Oral Investig..

[59] Ji E.Y., George K.A., Ahmadi Z., Bender E., Finkelman M., Mintline M., Parad S., Porto T., C. Sheng C., Tadinada A. (2026). Integrating Artificial Intelligence in Periodontal Diagnosis: A Comparative Evaluation of ChatGPT-4 and Dental Educators. J. Dent. Educ..

[60] Amugo I., Frederickson K.L., Rajakaruna H., Xie H., Gangula P., Shanker A., Wang Q. (2026). Evaluation of GPT-5 in Periodontitis Staging and Grading: Retrospective Observational Study. JMIR Form. Res..

[61] Ossowska A., Kusiak A., Camlet A., Świetlik D. (2026). Diagnostic Performance and Error Patterns of a Large Language Model and Neural Network in Periodontitis Classification: A Comparative Study. JCM.

[62] Holtfreter B., Kuhr K., Borof K., Tonetti M.S., Sanz M., Kornman K., Jepsen S., Aarabi G., Völzke H., Kocher T. (2024). ACES: A New Framework for the Application of the 2018 Periodontal Status Classification Scheme to Epidemiological Survey Data. J. Clin. Periodontol..

[63] Liu J., Liu F., Wang C., Liu S. (2025). Prompt Engineering in Clinical Practice: Tutorial for Clinicians. J. Med. Internet Res..

[64] Callens S. (2026). Effective Prompt Design for Large Language Models in Clinical Practice. Acta Clin. Belg..

[65] Wang J., Shi E., Yu S., Wu Z., Hu H., Ma C., Dai H., Yang Q., Kang Y., Wu J. (2026). Prompt Engineering for Healthcare: Methodologies and Applications. Meta-Radiology.

[66] Shah K., Xu A.Y., Sharma Y., Daher M., McDonald C., Diebo B.G., Daniels A.H. (2024). Large Language Model Prompting Techniques for Advancement in Clinical Medicine. J. Clin. Med..

[67] Butera A., Folini E., Cosola S., Russo G., Scribante A., Gallo S., Stablum G., Menchini Fabris G.B., Covani U., Genovesi A. (2023). Evaluation of the Efficacy of Probiotics Domiciliary Protocols for the Management of Periodontal Disease, in Adjunction of Non-Surgical Periodontal Therapy (NSPT): A Systematic Literature Review. Appl. Sci..

[68] Scribante A., Butera A., Alovisi M. (2022). Customized Minimally Invasive Protocols for the Clinical and Microbiological Management of the Oral Microbiota. Microorganisms.

[69] Oh S.-L., Yang J.S., Kim Y.J. (2021). Discrepancies in Periodontitis Classification among Dental Practitioners with Different Educational Backgrounds. BMC Oral Health.

[70] Abou-Arraj R.V., Kaur M., Alkhoury S., Swain T.A., Geurs N.C., Souccar N.M. (2021). The New Periodontal Disease Classification: Level of Agreement on Diagnoses and Treatment Planning at Various Dental Education Levels. J. Dent. Educ..

[71] Labrak Y., Rouvier M., Dufour R. (2023). A Zero-Shot and Few-Shot Study of Instruction-Finetuned Large Language Models Applied to Clinical and Biomedical Tasks. arXiv.

[72] Sivarajkumar S., Kelley M., Samolyk-Mazzanti A., Visweswaran S., Wang Y. (2024). An Empirical Evaluation of Prompting Strategies for Large Language Models in Zero-Shot Clinical Natural Language Processing: Algorithm Development and Validation Study. JMIR Med. Inform..

[73] Alazraki L., Chen L., Brassard A., Stacey J., Rahmani H.A., Rei M. (2026). AgentCoMa: A Compositional Benchmark Mixing Commonsense and Mathematical Reasoning in Real-World Scenarios. Proceedings of the Proceedings of the 64th Annual Meeting of the Association for Computational Linguistics (Volume 1: Long Papers).

[74] Press O., Zhang M., Min S., Schmidt L., Smith N., Lewis M. (2023). Measuring and Narrowing the Compositionality Gap in Language Models. Proceedings of the Findings of the Association for Computational Linguistics: EMNLP 2023.

[75] Dziri N., Lu X., Sclar M., Li X., Jiang L., Lin B.Y., Welleck S., West P., Bhagavatula C., Bras R.L. (2023). Faith and Fate: Limits of Transformers on Compositionality. Proceedings of the Advances in Neural Information Processing Systems 36.

[76] Kahl S., Löffler F., Maciol M., Ridder F., Schmitz M., Spanagel J., Wienkamp J., Burgahn C., Schilling M., Bellas F., Fontenla-Romero O. (2025). Evaluating the Impact of Advanced LLM Techniques on AI Lecture Tutors for a Robotics Course. AI in Education and Educational Research.

[77] Lu Z., Tian J., Wei W., Qu X., Cheng Y., Xie W., Chen D. (2024). Mitigating Boundary Ambiguity and Inherent Bias for Text Classification in the Era of Large Language Models. arXiv.

[78] Chen T., Li P., Zhou K., Chen T., Wei H. (2025). Unveiling Privacy Risks in Multi-Modal Large Language Models: Task-Specific Vulnerabilities and Mitigation Challenges. Proceedings of the Findings of the Association for Computational Linguistics: ACL 2025.

[79] López-Úbeda P., Martín-Noguerol T., Luna A. (2026). Environmental and Economic Costs behind LLMs. Int. J. Comput. Assist. Radiol. Surg..

[80] Gadbail S., Desai M., Karlapalem K. (2025). Iti-Validator: A Guardrail Framework for Validating and Correcting LLM-Generated Itineraries. Proceedings of the Proceedings of the 33rd ACM International Conference on Advances in Geographic Information Systems.

[81] Ghaffarzadeh-Esfahani M., Ghaffarzadeh-Esfahani M., Salahi-Niri A., Toreyhi H., Atf Z., Mohsenzadeh-Kermani A., Sarikhani M., Tajabadi Z., Shojaeian F., Bagheri M.H. (2025). Large Language Models versus Classical Machine Learning Performance in COVID-19 Mortality Prediction Using High-Dimensional Tabular Data. Sci. Rep..

[82] Kefeli J., Berkowitz J., Acitores Cortina J.M., Tsang K.K., Tatonetti N.P. (2024). Generalizable and Automated Classification of TNM Stage from Pathology Reports with External Validation. Nat. Commun..

[83] Singhal K., Tu T., Gottweis J., Sayres R., Wulczyn E., Amin M., Hou L., Clark K., Pfohl S.R., Cole-Lewis H. (2025). Toward Expert-Level Medical Question Answering with Large Language Models. Nat. Med..

[84] Wu C., Lin W., Zhang X., Zhang Y., Xie W., Wang Y. (2024). PMC-LLaMA: Toward Building Open-Source Language Models for Medicine. J. Am. Med. Inform. Assoc..

[85] Luo R., Sun L., Xia Y., Qin T., Zhang S., Poon H., Liu T.-Y. (2022). BioGPT: Generative Pre-Trained Transformer for Biomedical Text Generation and Mining. Brief. Bioinform..

